# WDR62-deficiency Causes Autism-like Behaviors Independent of Microcephaly in Mice

**DOI:** 10.1007/s12264-022-00997-5

**Published:** 2022-12-26

**Authors:** Dan Xu, Yiqiang Zhi, Xinyi Liu, Le Guan, Jurui Yu, Dan Zhang, Weiya Zhang, Yaqing Wang, Wucheng Tao, Zhiheng Xu

**Affiliations:** 1 Fujian Key Laboratory of Molecular Neurology, Institute of Neuroscience, Fujian Medical University, Fuzhou, 350005 China; 2 College of Biological Science and Engineering, Institute of Life Sciences, Fuzhou University, Fuzhou, 350108 China; 3 University of Chinese Academy of Sciences, Beijing, 100101 China; 4 State Key Laboratory of Molecular Developmental Biology, CAS Center for Excellence in Brain Science and Intelligence Technology, Institute of Genetics and Developmental Biology, Chinese Academy of Sciences, Beijing, 100101 China; 5 Key Laboratory of Brain Aging and Neurodegenerative Diseases, School of Basic Medical Sciences, Fujian Medical University, Fuzhou, 350108 China

**Keywords:** WDR62, Microcephaly, Autism spectrum disorder, Synapse, Retinoic acid

## Abstract

Brain size abnormality is correlated with an increased frequency of autism spectrum disorder (ASD) in offspring. Genetic analysis indicates that heterozygous mutations of the WD repeat domain 62 (*WDR62*) are associated with ASD. However, biological evidence is still lacking. Our study showed that *Wdr62* knockout (KO) led to reduced brain size with impaired learning and memory, as well as ASD-like behaviors in mice. Interestingly, *Wdr62* Nex-cKO mice (depletion of WDR62 in differentiated neurons) had a largely normal brain size but with aberrant social interactions and repetitive behaviors. WDR62 regulated dendritic spinogenesis and excitatory synaptic transmission in cortical pyramidal neurons. Finally, we revealed that retinoic acid gavages significantly alleviated ASD-like behaviors in mice with WDR62 haploinsufficiency, probably by complementing the expression of ASD and synapse-related genes. Our findings provide a new perspective on the relationship between the microcephaly gene *WDR62* and ASD etiology that will benefit clinical diagnosis and intervention of ASD.

**Supplementary Information:**

The online version contains supplementary material available at 10.1007/s12264-022-00997-5.

## Introduction

Microcephaly is usually caused by defects in brain development and is often accompanied by intellectual disability and other neurological issues, such as seizures and sensorineural hearing loss [1]. To date, at least 28 genes have been identified as causative genes of autosomal recessive primary microcephaly (MCPH), ranging from *MCPH1* to *MCPH28*. Mutants of most of them have been established in animal models, such as zebrafish, *Drosophila*, mouse, and ferret [2]; however, behavioral analyses of these animal models are lacking. WD-repeat domain 62 (*WDR62*) has been identified as the second most causative gene of MCPH [3–5]. Clinical studies in patients with *WDR62* mutations have reported abnormal behavioral phenotypes, including impaired learning and spatial memory, speech and motor delays, and seizures [6–8].

The phenotype-to-genotype approach has revealed head-circumference-associated genes in an autism spectrum disorder (ASD) cohort [9]. Targeted sequencing and functional analysis have revealed that some microcephaly or macrocephaly risk genes are involved in ASD [10]. *WDR62* is associated with intellectual disability and is listed as a high-risk gene for autism based on six support vector machine classifiers trained using different types of control gene lists [11]. Whole-genome sequencing of 32 Chinese parent-offspring trios with ASD showed several microcephaly-associated genes, which included *WDR62* with rare *de novo* mutations [12]. These data indicate that *WDR62* is a possible candidate gene for ASD.

Retinoic acid (RA), a hormone derivative of vitamin A, is considered to be a potent mediator of homeostatic synaptic plasticity in the human cortex [13]. Increasing evidence suggests that vitamin A deficiency exacerbates autism-like behaviors and other cognitive disorders [14, 15]. Evidence from both human and animal studies indicates that RA homeostasis and the disruption of RA signaling are associated with ASD [16, 17]. Therefore, Vitamin A or RA supplementation has been shown to alleviate social impairment in various animal models and children with ASD [16, 18–20]. Our previous studies indicate that WDR62 is required for RA-induced meiotic gene expression in germ cells [21]. However, whether RA is involved in WDR62-mediated brain development and neurological diseases is currently unclear.

Here, we engineered two *Wdr62*-knockout (KO) mice: *Wdr62*^flox/flox^; ZP3-Cre (*Wdr62*^*−/−*^*, Wdr62-* KO), which drives WDR62 deletion in the germline, and *Wdr62*^flox/flox^; Nex-Cre (*Wdr62*-Nex-cKO), which leads to deletion of *WDR62* in early post-mitotic excitatory neurons of the developing forebrain. *Wdr62*-KO mice, but not *Wdr62*-Nex-cKO mice showed reduced brain size. Interestingly, behavioral examinations indicated that both kinds of mice displayed abnormal sociability. Furthermore, morphological and electrophysiology analyses of pyramidal neurons indicated a synaptic development defect and plasticity dysfunction in *Wdr62*-deficient neurons. Finally, we found that oral gavage of RA significantly reverses ASD-like phenotypes in *Wdr62*-deficient mice.

## Materials and Methods

### Animals

All animal procedures used in this study were performed according to protocols approved by the Institutional Animal Care and Use Committee at the Institute of Genetics and Developmental Biology, Chinese Academy of Sciences (protocol number: AP2016053), and the Institutional Animal Care and Use Committee of Fujian Medical University (protocol number: FJMUIACUC 2021-J-0164). Details are described in the Supplement.

### Antibodies

The antibodies used for western blotting were as follows: GAPDH (CST, 2118s, 1:2,000) and WDR62 antibody generated by MBL Co. using the antigen VGQGGNQPKAGPLRAGTC. The antibodies used for immunostaining were NeuN (Abcam, ab104224, 1:1000) and PV (Sigma, P3088, 1:1000). Nuclei were stained with DAPI (4′,6-diamidino-2-phenylindole) (Invitrogen).

### Histology, Immunofluorescence Staining, and Imaging

For cryosections, adult (2 months old) mouse brains were fixed in 4% paraformaldehyde solution, cryoprotected in 30% sucrose, and frozen in the tissue-freezing medium. Sections 40–50 μm thick were cut for immunofluorescence staining as described previously [22]. To analyze *in vivo* dendritic arborization and spine morphology, Thy1-GFP-positive age-matched littermates were perfused at 8 weeks of age, and 100-μm coronal sections were cut on a freezing microtome and immunostained with anti-GFP. Sections were imaged on an LSM 780 (Carl Zeiss) or FV3000 (Olympus) confocal microscope. Large-scale images were captured by an Olympus Slideview VS200. All of the images were analyzed with ImageJ, OlyVIA, or Imaris software.

### Nissl Staining

Two-month-old adult mouse brain slices were stained with 0.1% toluidine blue in 30 min for Nissl bodies. They were dehydrated in turn by 70% alcohol for 45 s, 96% alcohol for 45 s, and 99% alcohol for 45 s, twice each, then the slices were cleared in xylene for >30 min. The resin was used for mounting.

### Transmission Electron Microscopy

One-month-old Ctrl and *Wdr62*-Nex-cKO mice were perfused with 4% PFA (pH 7.4). The primary somatosensory cortex was dissected and post-fixed with 2.5% glutaraldehyde in phosphate buffer (PB) at 4°C overnight, washed with PBS, post-fixed with 1% osmium tetroxide in PB for 1 h at room temperature, rinsed with PBS, stained with 2% uranyl acetate in 70% ethanol at 4°C for 1 h, dehydrated with serial dilutions of ethanol, incubated in propylene oxide, and embedded in Epon resin. Tissues were cut at 65 nm on an ultramicrotome (Leica, UC6) and counterstained with uranyl acetate and lead citrate. Grids were viewed on an electron microscope (Tecnai G2J) and digital images were captured with a CCD camera.

### Culture of Mouse Embryonic Fibroblasts and Western Blotting

Mouse embryonic fibroblasts (MEFs) were isolated at E12.5. The embryonic skin was dissociated and then trypsinized to produce single-cell suspensions. Cells were cultured in DMEM+10% FBS+1% PS. Western blotting was performed as previously described [23]. To detect endogenous WDR62, lysates were prepared from cultured MEFs or mouse brains of different genotypes using lysis buffer containing 50 mmol/L Tris-HCl pH 7.4, 150 mmol/L NaCl, 1% NP40, 0.5% DOC, 1 mmol/L EDTA, and 2 mmol/L PMSF. Cell debris was pelleted at 12,500 rpm for 10 min at 4 °C and the supernatant was collected.

### Behavioral Study

All mice were housed under the same conditions for the behavioral tests. Age-matched mice were subjected to behavioral assays in the Mouse Behavioral Assessment, as performed by [24, 25], with subtle modifications. All mice used for behavioral tests were 8–12 week-old littermates with comparable body weights. Autism-associated behaviors were assessed first, then learning-related tasks, and last the motor behaviors. Each cohort of mice was given at least 3 days’ break between tasks. All behavioral tests were conducted by trained experimentalists blinded to animal genotypes. Handling and specific tests are described in the Supplement.

### Acute Slice Preparation and Whole-cell Recording

Procedures are described in the Supplementary Material.

### RNA sequencing and Data analysis

Procedures are described in the Supplementary Material.

### Quantification and Statistical Analysis

Statistical analysis was carried out using GraphPad Prism 8.0.2 (GraphPad Software, La Jolla, CA, USA). For most data, the Gaussian distribution of the data was assessed using the D’Agostino and Pearson normality and Shapiro-Wilk normality tests. If the data passed the Gaussian distribution test, parametric unpaired two-tailed t-tests were used for two groups and one-way analyses of variance (ANOVA), followed by Tukey’s multiple comparisons tests, for three or more groups. Otherwise, nonparametric unpaired Mann–Whitney tests were used for two groups. Two-way ANOVAs with Bonferroni’s multiple comparisons test was used for experiments containing two independent variables. Data were analyzed blinded to the experimental conditions.

## Results

### Wdr62-KO Mice Mimic Human Microcephalic Hypophrenia

**Fig. 1:**
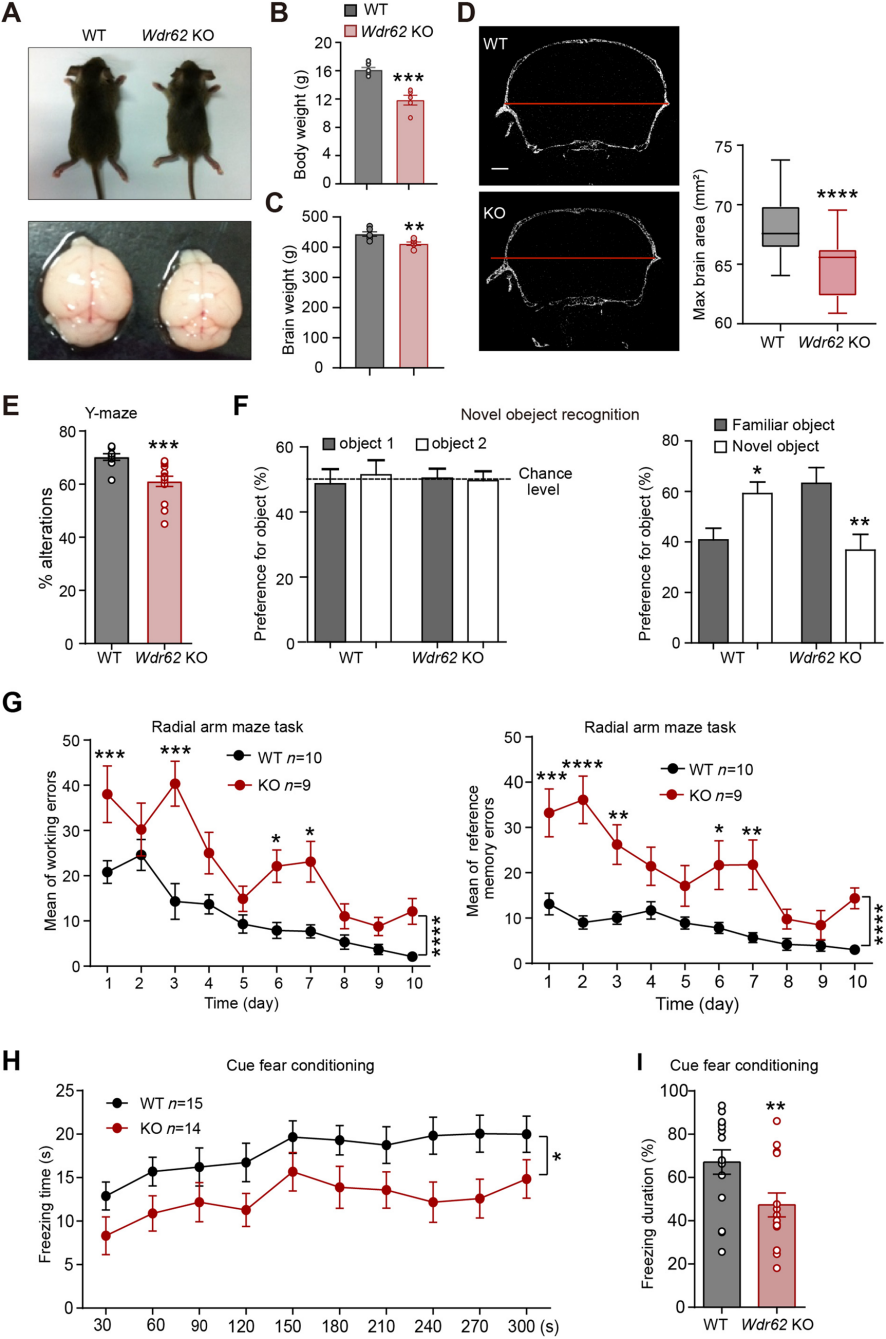
*Wdr62* KO mice mimic human microcephalic hypothermia**. A** Images of *Wdr62*-KO mice and WT littermates and their brains at P20. **B, C** Body and brain weights of 1-month-old *Wdr62*-KO and WT littermate mice (WT *n =* 5, *Wdr62*-KO *n =* 6). **D** Left: CT images of 4-month-old WT and KO mouse brains. Right: quantification of average maximal brain areas in D (30 brain slices from 3 WT mice and 31 brain slices from 3 *Wdr62*-KO mice). **E** Y maze spontaneous alternation test. *Wdr62-*KO mice show decreased spontaneous alternation (WT *n =* 14, KO *n =* 18). **F** Novel objection recognition. Left: time spent exploring the original two objects in 5 min does not differ significantly between WT and KO mice. Right: KO mice spend significantly more time sniffing the familiar object, whereas the WT mice show more curiosity for the novel object (WT *n =* 11, KO *n =* 10). **G** Radial arm maze test. Working memory and reference memory errors were recorded daily. *Wdr62-*KO mice show deficiencies in spatial learning and memory (WT *n =* 10, KO *n =* 9).** H** Cue fears conditioning test. Freezing time was measured during the 5-min test session 48 h after training. **I** Freezing duration during the cue fear conditioning test assesses fear memory. [(**H**. **I**) WT *n =*15, KO *n =*14]. All data are presented as the mean ± SEM; ***P <0.001; **P <0.01; *P <0.05; ns *P* >0.05. Two-tailed unpaired *t*-tests (**A–D, F, I**), Mann-Whitney test (**E**), two-way ANOVAs with Bonferroni’s multiple comparisons tests (**G** and **H**). Scale bar, 1 mm (**D**).

We and others have identified the roles of WDR62 in neural progenitor proliferation and differentiation during embryonic brain development [26, 27]. To explore the function of WDR62 during the postnatal stage, we engineered *Wdr62*-KO mice as described previously [21] by crossing *Wdr62*^flox/+^ with ZP3-Cre transgenic mice. The *Wdr62*-KO mice showed a normal lifespan but infertility, similar to gene trap mice reported previously [27, 28]. In addition, *Wdr62-*KO mice showed dwarfism and a significant reduction in body and brain weight at one-month-old (Fig.1A–C). Micro-computed tomography analysis confirmed the significantly reduced head size in adult mice which mimicked microcephaly (Fig.1D). The null allele was confirmed in the brain and MEFs using quantitative PCR and western blot analysis (Fig. S1A–C). Further, the initial characterization of *Wdr62*-KO mouse brain with Nissl staining did not reveal other morphological defects (Fig.S1D).

Intellectual disability is one of the most important symptoms of microcephaly patients. To assess the learning and memory of *Wdr62-*KO mice, we applied the Y maze and novel object recognition tests in *Wdr62*-KO mice and wild-type (WT) littermates. During the 5-min Y maze test, the percentage of alternation was significantly lower in KO mice than in WT mice (Fig. 1E). In the novel object recognition task, both genotypes explored the two identical objects similarly during the automated familiarization phase. However, the *Wdr62-*KO mice spent less time exploring the unfamiliar object, which indicated a memory deficit (Fig. 1F). Learning performance was further evaluated using the four-arm baited version of the eight-arm radial maze test. Consistently, the *Wdr6*2-KO mice made more working memory and reference memory errors after training than WT mice (Fig. 1G). In addition, fear conditioning (FC) was applied to examine non-spatial passive avoidance learning. There was no genotype-dependent statistical difference in the startle response in the contextual FC. However, 48 h later, the mice were placed into the box with a new context and sound, and the *Wdr6*2-KO mice showed a significantly shorter freezing duration than WT mice in the cue FC (Fig. 1H, I). These results indicated that *Wdr6*2-KO mice had impairments in learning and memory that mimic the mental decline in human microcephaly patients.

### Wdr62-KO Mice Display Reduced Social Interaction

**Fig. 2:**
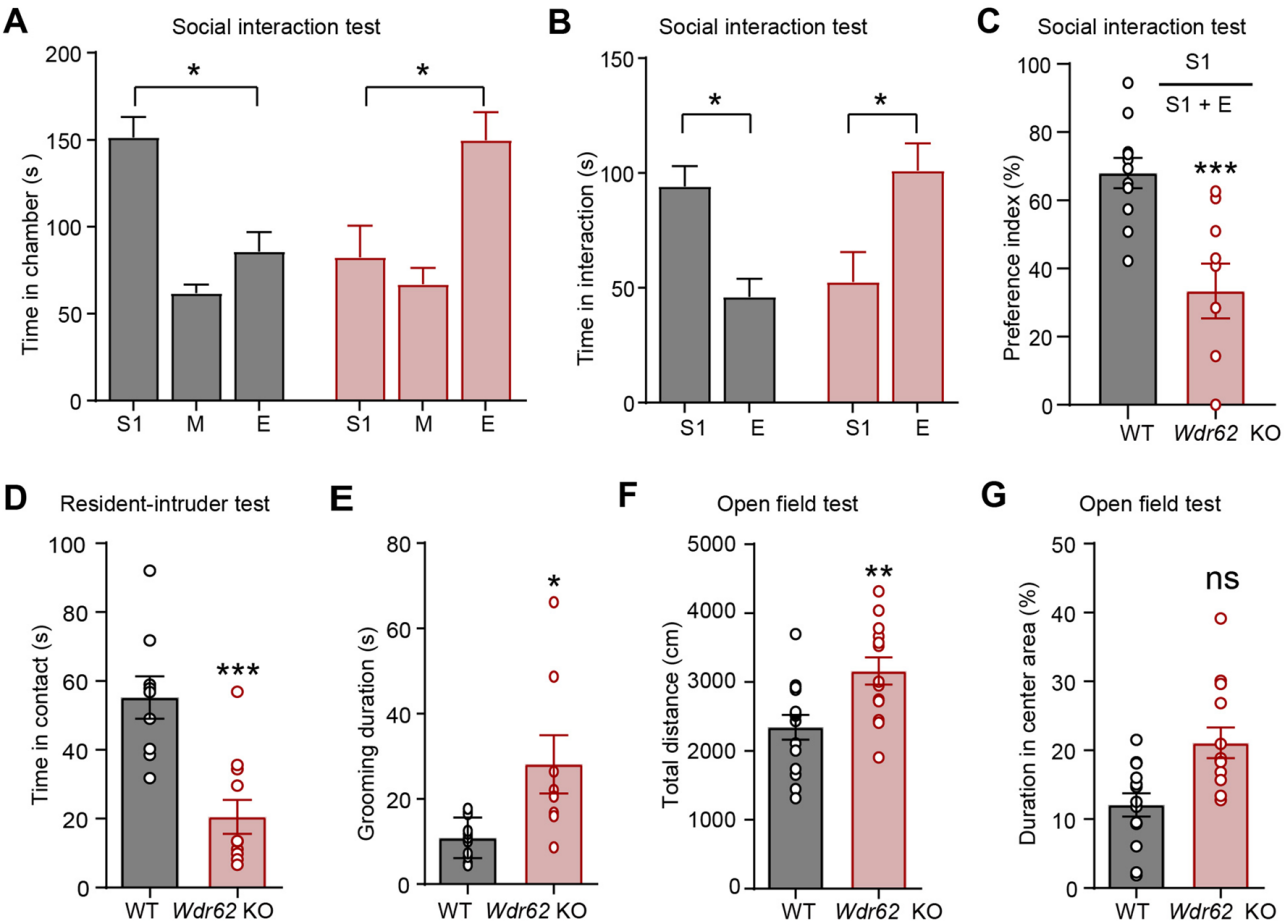
*Wdr62*-KO mice exhibit ASD-related behavior. **A** *Wdr62*-KO mice spend less time in the chamber containing the Stranger 1 (S1) mouse and more time in the chamber containing the empty cage (**E**) than WT mice (WT *n =* 10, KO *n =* 9). **B** Time spent sniffing S1 or E. **C** Graph showing the social preference index [S1/(S1+E)]. **D** *Wdr62-*KO exhibited reduced social interaction during the home cage social interaction test (WT *n =* 11, KO *n =* 9).** E** *Wdr62-*KO mice spend more time than WT mice in self-grooming (WT *n* =13, KO *n =* 8). **F** Quantification of the total distance traveled during the 15-min open field test (OFT; WT *n =* 15, KO *n =* 17). **G** Quantification of relative time spent in the center area in the OFT (WT *n =* 15, KO *n =* 17). All data are presented as the mean ± SEM; ***P <0.001; **P <0.01; *P <0.05; ns *P >*0.05. Mann-Whitney test (**A** and **B**), two-tailed unpaired *t*-test (**C–G**).

Because *de novo* mutation of *Wdr6*2 has been found in ASD patients and ~15% of ASD patients have microcephaly [10, 29, 30], we went on to inspect whether *Wdr62-*KO mice display autistic behaviors. Animals were subjected to the three-chamber test which is often used to identify deficits in sociability and/or social novelty. During the social interaction session, WT mice preferred to spend significantly more time in the chamber with the social partner, stranger 1 (S1) than in the empty chamber (E), whereas *Wdr62-*KO littermates showed no apparent preference (Fig. 2A, B). *Wdr62-*KO mice also displayed a markedly reduced social preference index for S1 (Fig. 2C). In addition, during the home-cage social interaction test, WT mice exposed to a novel nonspecific juvenile exhibited typical behaviors of approaching and sniffing. However, such initial social interactions were significantly fewer for *Wdr62* KO mice (Fig. 2D). Individuals with autism have repetitive behavior. We observed a significant increase in repetitive self-grooming in *Wdr62-*KO mice (Fig. 2E). Hyperactivity is also a common phenomenon with social deficits in mice [31, 32]. The open field test (OFT) is often used to measure exploratory behavior and general activity. We found that *Wdr62*-KO mice ran a much longer distance than WT mice did within the 15 min test period (Fig. 2F), however, there was no significant difference in the center distance (Fig. 2G). These results indicated that *Wdr62*-KO mice exhibited hyperactivity without apparent anxiety behavior. Taken together, our results revealed that *Wdr6*2-KO mice display certain aspects of ASD-like behaviors.

### Mice with Wdr62 Deletion in Post-mitotic Neurons Show Autism-like Behaviors but Largely Normal Brain Size and Learning/Memory

**Fig. 3:**
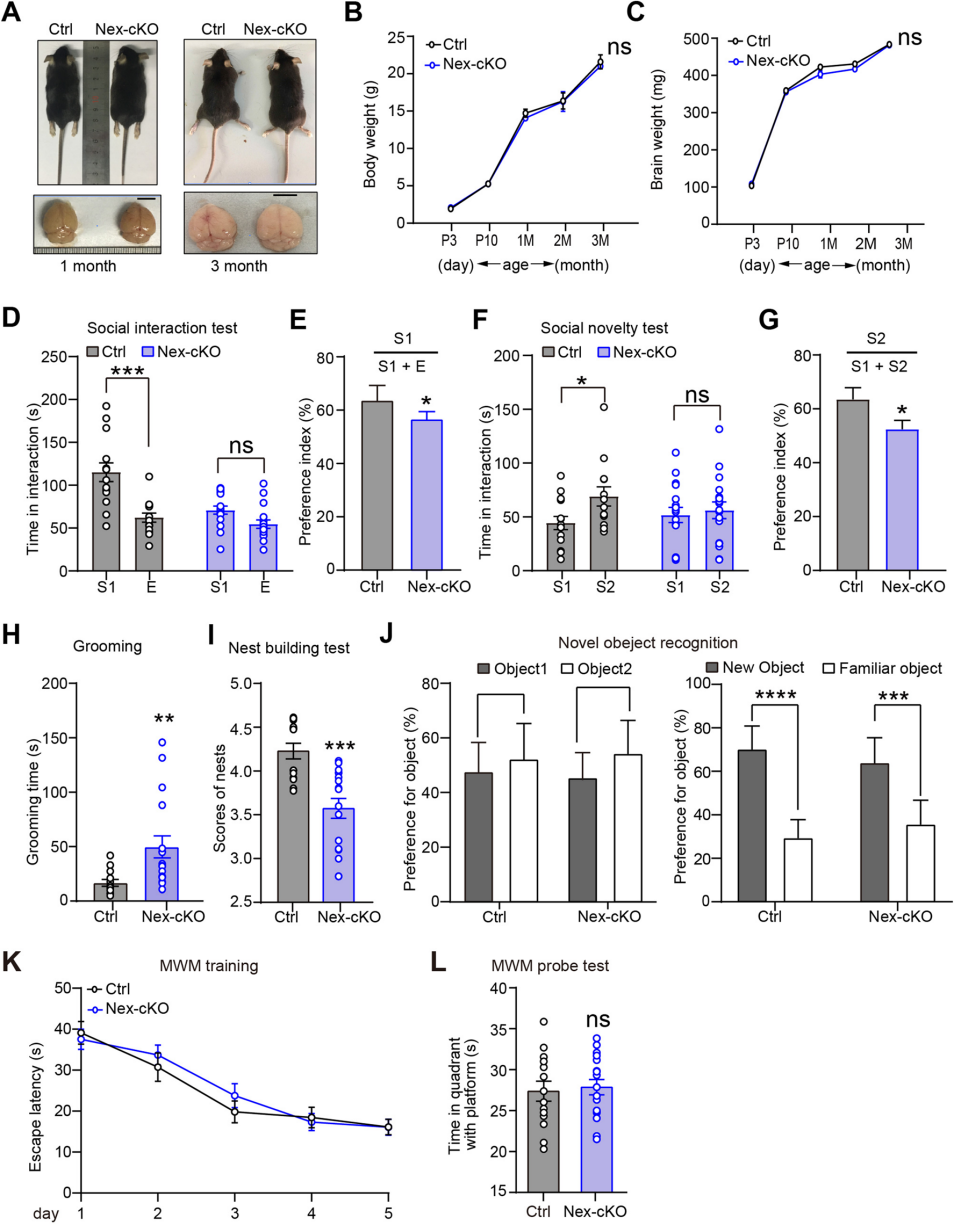
Mice with *Wdr62* deficiency in postmitotic neurons display ASD-related behaviors**. A** Images of *Wdr62-*Nex-cKO mice and littermate controls (Ctrl) and their brains at 1 month (left) and 3 months (right) of age show normal body and brain size. **B, C** Body/brain weights of *Wdr62-*Nex-cKO and Ctrl mice at different developmental stages. For both B and C, P3: Ctrl *n =* 6, *Wdr62-*Nex-cKO *n =* 8; P10: Ctrl *n =* 8, *Wdr62-*Nex-cKO *n =* 7; 1 month: Ctrl *n =* 5, *Wdr62-*Nex-cKO *n =* 5; 2 months: Ctrl *n =* 3, *Wdr62-*Nex-cKO *n =* 3; 3 months: Ctrl *n =* 6, *Wdr62-*Nex-cKO *n =* 6. **D** *Wdr62-*Nex-cKO mice spend less time interacting with social partner 1 (S1) than Ctrl mice. **E** Graph showing a significantly lower preference index for S1 of *Wdr62*-Nex-cKO mice than Ctrl mice in the social interaction test. **F** In the social novelty test, *Wdr62*-Nex-cKO mice display no preference for interacting with the S2. **G** The preference index of social novelty (% of time spent investigating S2 out of total object investigation time) is significantly reduced in *Wdr62*-Nex-cKO mice.** H** *Wdr62*-Nex-cKO mice spend significantly more time self-grooming in 10 min than Ctrls. **I** The nest-building test indicated significantly impaired nesting behavior in *Wdr62*-Nex-cKO mice. **J** Novel object recognition test. **K, L** The Morris water maze test indicates normal learning and memory in *Wdr62*-Nex-cKO mice. All data are presented as the mean ± SEM; ****P <0.0001; ***P <0.001; **P <0.01; *P <0.05; ns: *P >*0.05. (**D–L**) Ctrl *n =* 14, *Wdr62-*Nex-cKO *n =* 17. Two-way ANOVAs with Bonferroni’s multiple comparisons tests (**B–D** and **F**), two-tailed unpaired *t*-test (**G**), Mann-Whitney tests (**E**, **H**, and **I**). Scale bar, 1 cm (**A**).

Around 15% and 25% of ASD patients have either microcephaly or macrocephaly, respectively [29, 30], and we have shown above that *Wdr6*2-KO mice display certain aspects of ASD-like behavior [10, 12]. The social interaction defects in *Wdr62*-KO mice may be caused by reduced brain size. To test this postulate, we constructed *Wdr62*-Nex-cKO mice to delete WDR62 in post-mitotic neurons. The mRNA expression profiles indicated significantly reduced *Wdr62* expression at the postnatal stage when most neural progenitor cells had differentiated into neurons (Fig. S2A). We monitored the body and brain weight of *Wdr62-*Nex-cKO mice from postnatal day 3 (P3) to 3 months old. In contrast to *Wdr62-*KO mice, conditional knockout of *Wdr62* using Nex CRE had no evident effect on postnatal body and brain weight (Fig. 3A–C). Neuronal nuclear protein (NeuN) staining of P3 mouse brains showed that *Wdr62*-Nex-cKO mice had largely normal cortical thickness (Fig. S2B). Nevertheless, *Wdr62*-Nex-cKO mice showed impaired social interaction and social novelty compared with control mice (*Wdr62*^flox/flox^, Ctrl) in the analysis of the three-chamber test (Fig. 3D–G). The abnormal social behaviors were not due to impaired olfactory function as revealed by the comparable performance of the Ctrl and Nex-cKO mice in the buried food pellet test (Fig. S3A). In addition, *Wdr62*-Nex-cKO mice spent significantly more time grooming themselves than Ctrl mice (Fig. 3H). Nest building is often used to assess well-being and social and mating behaviors. Using pressed cotton squares and a definitive five-point nest-rating scale [33], we found a significantly lower nesting score in *Wdr62*-Nex-cKO mice than in Ctrl mice (Fig. 3I). Together, our results indicated that WDR62 deficiency in post-mitotic neurons also leads to autism-like behaviors in mice.

We went on to inspect whether *Wdr62*-Nex-cKO mice had deficits in learning and memory. In contrast to *Wdr62*-KO mice, there were no significant differences between *Wdr62*-Nex-cKO mice and their littermate controls in the novel recognition test and the Morris water maze test, an indication of normal spatial learning and memory (Fig. 3J–L). However, *Wdr62*-Nex-cKO mice showed a slightly impaired short-term memory in the Y maze test (Fig. S3B). Notably, *Wdr6*2-KO mice showed hyperactivity in the OFT (Fig. 2F), but *Wdr62*-Nex-cKO mice showed no significant differences in total distance and time in the center area compared with control mice (Fig. S3C). In addition, *Wdr62*-Nex-cKO mice had no defects in locomotor activity during the rotarod test (Fig. S3D). Moreover, *Wdr62*-Nex-cKO mice showed no differences in times of entry into the open arm in the elevated plus-maze test, which indicated normal anxiety susceptibility after WDR62 deletion in post-mitotic neurons (Fig. S3E). In summary, WDR62 deficiency in post-mitotic neurons resulted in impaired social interactions and stereotyped behaviors in mice, without significantly affecting the size of the brain, learning, memory, or locomotor activity.

### WDR62 Regulates Neuronal Dendritic Complexity and Spine Density in Cortical Pyramidal Neurons

**Fig. 4:**
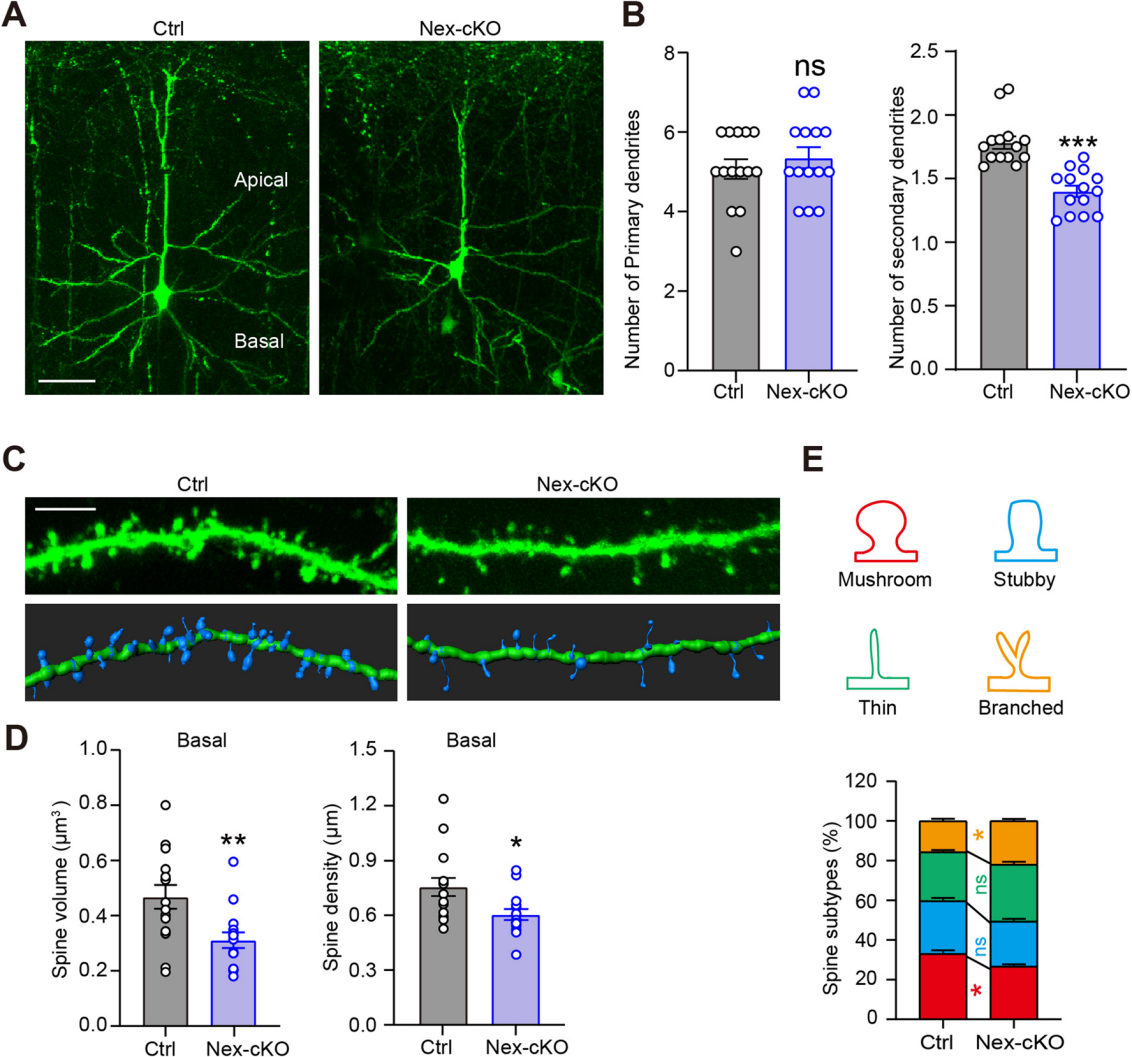
WDR62 regulates dendritic arborization and the spinogenesis of pyramidal neurons. **A** Representative images of Thy1-GFP*m*-labeled layer 2/3 pyramidal neurons in 2-month-old control (Ctrl) and *Wdr62*-Nex-cKO mouse cortices. **B** Quantification indicates reduced secondary dendritic arborization in *Wdr62*-deficient neurons (Ctrl mice: 14 neurons in three mice; *Wdr62*-Nex-cKO mice: 14 neurons in 3 mice). **C** Higher-magnification images of basal dendritic spines from Thy1-*GFPm*-labeled secondary dendrites of layer 2/3 pyramidal neurons in the primary cortex. **D** Quantification of total basal spine volume/density in **C**. Ctrl and *Wdr62*-Nex-cKO mice (*n =* 15 neurons from 3 mice). **E** The proportions of basal dendritic spines that were mushroom (mature), stubby (mature), thin (immature), and branched (immature) subtypes; (*n =* 15 neurons from 3 independent mice. All data are presented as means ± SEM; ***P <0.001; **P <0.01; *P <0.05; ns *P >*0.05. Two-way ANOVA with Bonferroni’s multiple comparisons test (**E**), two-tailed unpaired *t*-tests (**B** and **D**). Scale bar, 50 μm (**A**) and 5 μm (**C**).

Structural and functional alterations of the primary somatosensory cortex have been reported to regulate social behavior, socio-emotional processing, and affective functions in humans and preclinical animal models [34–37]. To investigate the cellular mechanisms underlying abnormal behaviors in *Wdr62-*Nex-cKO mice, we analyzed pyramidal neuronal morphology by crossing *Wdr62-*Nex-cKO mice with Thy1-*GFPm* transgenic mice, which express EGFP in sparse subsets of excitatory neurons [38]. Compared with Ctrl littermates, the global organization of *Wdr62*-Nex-cKO mouse brains appeared largely normal (Fig. S4A). In the cortex, most GFP-expressing neurons were located in layer 5 of the motor cortex and a few in layer 2/3 of the somatosensory cortex (Fig. S4B). Morphological analysis revealed reduced numbers of secondary dendritic arborizations in the layer 2/3 pyramidal neurons of the somatosensory cortex in *Wdr62*-Nex-cKO mice (Fig. 4A). Motor cortex deficiency has also been implicated in the pathogenesis of ASD [39]. We found a consistent reduction in the dendritic arborization of layer 5 motor neurons (Fig. S5A). The reduced number of secondary dendritic branching in the dendrites of pyramidal neurons indicated decreased neuronal complexity in *Wdr62*-Nex-cKO mice compared with that in control mice (Figs 4B, S5B).

Dendritic spine development is a major cellular process that is disrupted in ASD [40]. Thus, we investigated whether WDR62 deficiency influences the density and/or morphology of spines and synapses. The spine density of layer 2/3 pyramidal neurons was significantly lower in the *Wdr62*-Nex-cKO mice than in the control group (Fig. 4C, D). In line with this, we also found significantly lower spine density in both apical and basal dendrites of layer 5 pyramidal motor neurons in *Wdr62*-Nex-cKO mice (Fig. S5C, D). In addition, different types of spine analysis indicated that the proportion of mature spines (stubby and mushroom) was reduced in *Wdr62*-Nex-cKO mice, whereas that of immature spines (branched and thin) was increased (Figs 4E, S5E). Similarly, the volumes rather than the lengths of spines decreased significantly in both apical and basal dendrites of layer 5 pyramidal neurons in *Wdr62*-Nex-cKO mice when compared with those in control mice (Fig. S5F, G). These results indicate that WDR62 is important for dendrite growth and dendritic spine development in cortical pyramidal neurons.

**Fig. 5:**
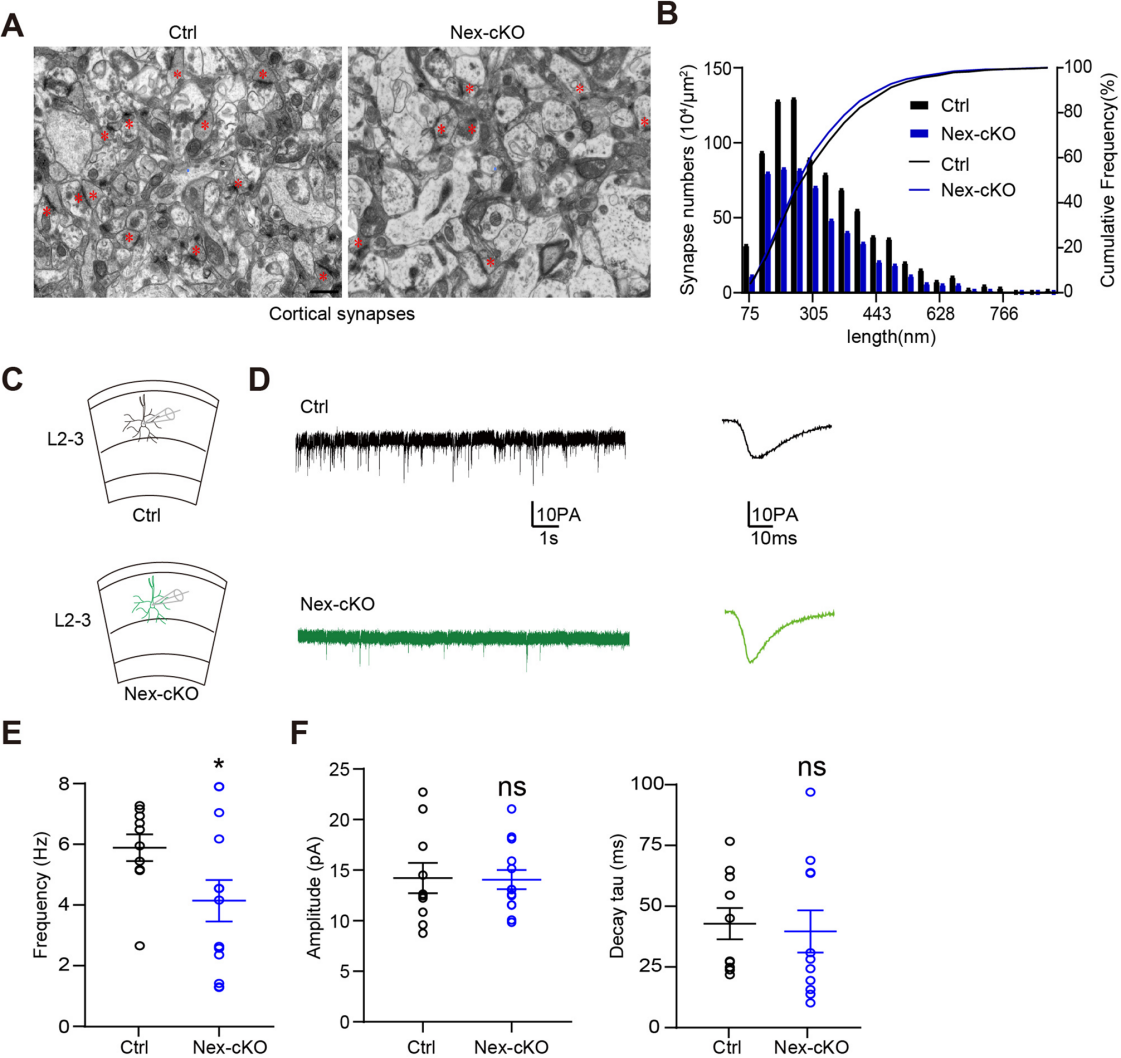
Reduced numbers of asymmetric synapses and disturbed synaptic plasticity in the *Wdr62-*deficient cortex. **A** Representative electron micrographs of asymmetric synapses (stars) in 1-month-old Ctrl and *Wdr62*-Nex-cKO mouse cortices. **B** Quantification showing reduced numbers of asymmetric synapses in *Wdr62-*Nex-cKO mouse cortices. **C** Schematic of mEPSC recordings from layer 2/3 pyramidal neurons. **D** Representative mEPSC traces in Ctrl and *Wdr62*-Nex-cKO neurons. **E–G** Summary data of mEPSC recordings (frequencies, amplitudes, and decay tau) in cortical slices from P17–P21 Ctrl and *Wdr62*-Nex-cKO mice (Ctrl *n =* 10 cells from 3 mice, *Wdr62*-Nex-cKO, *n =* 13 cells from two mice). All data are presented as the mean ± SEM; ***P <0.001; **P <0.01; *P <0.05; ns *P >*0.05. Two-tailed unpaired *t*-test (**E–G**). Scale bar, 1 μm (**A**).

To further explore the role of WDR62 in synapse development, we applied transmission electron microscopy analysis and revealed that the number, rather than the length, of asymmetric synapses (identified by the presence of a postsynaptic density), was significantly reduced in the cortex of 1-month-old *Wdr62*-Nex-cKO mice compared with that of control mice (Fig. 5A, B). Decreased dendritic complexity and spine density may cause the downregulation of neuronal activity. To examine the functional consequences of abnormal synaptic morphology/number on synaptic properties, we measured the frequency and amplitude of mEPSCs in layer 2/3 pyramidal neurons in acute cortical brain slices (Fig. 5C, D). Compared with littermate control mice, mEPSC frequency was significantly reduced in layer 2/3 pyramidal neurons of P17-P21 Nex-cKO mice, with no overt alterations of the mEPSC amplitude (Fig. 5E–G). The reduction in mEPSC frequency recorded in the postsynaptic *Wdr62* Nex-cKO cells was consistent with the alterations in synapse density. However, the change in dendritic spine maturation did not affect the mEPSC amplitude, which implied that the postsynaptic receptors might not be disturbed in *Wdr62* Nex-cKO mice.

### Oral Gavage of RA Ameliorates Social Interaction Deficits in Mice with Wdr62 Haploinsufficiency

**Fig. 6:**
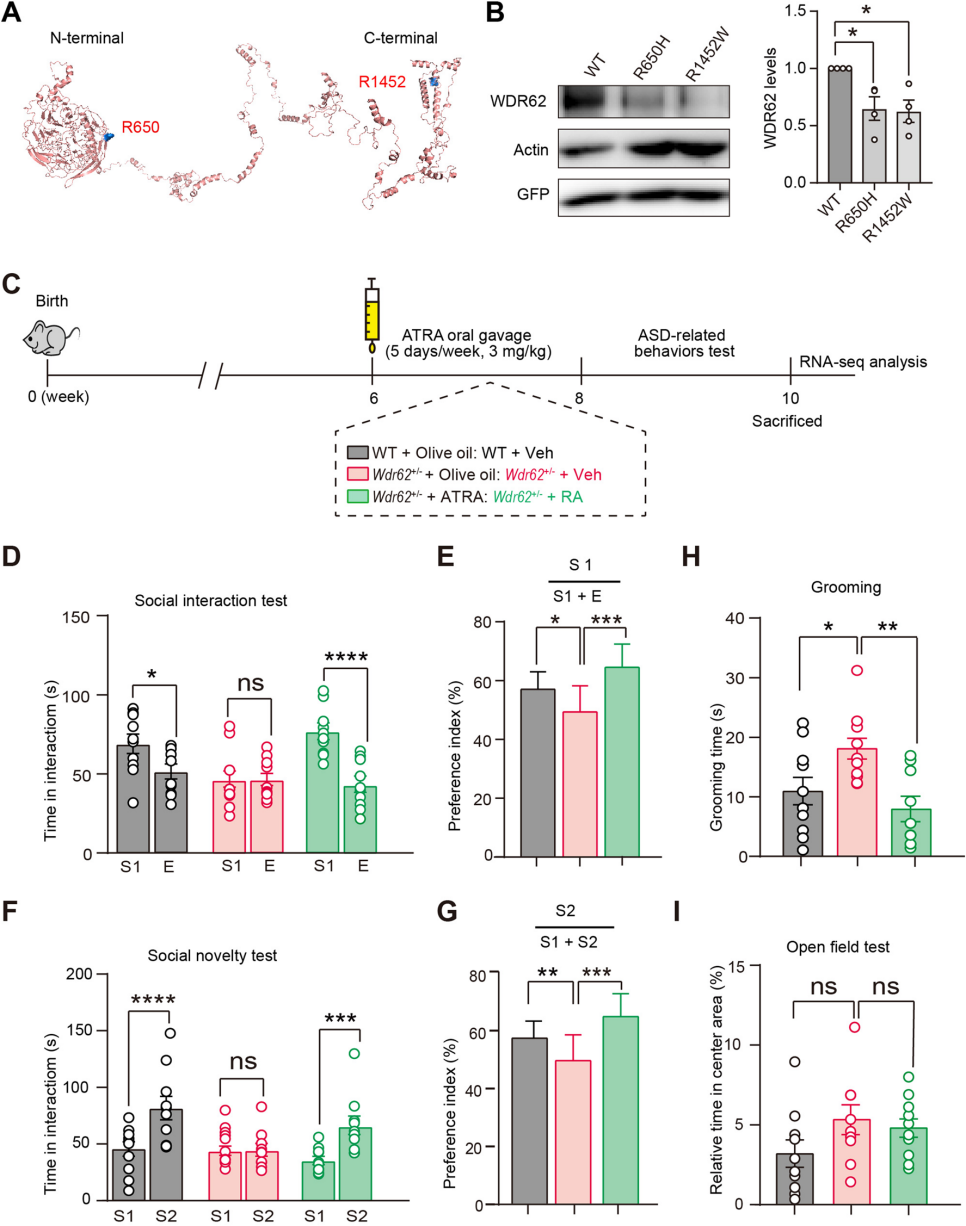
RA supplement reverses autistic-like phenotypes in *Wdr62*^+/−^ mice. **A** Three-dimensional PyMOL viewer of the WDR62 structure predicted by the protein structure database and the positions of ASD-related mutations. **B** Western blots of proteins from HEK 293T cells, transfected with WT and mutant pCMSEGFP-WDR62 plasmids. GFP was used to measure transfection efficiency and actin served as an internal Ctrl. The bar graph shows the protein expression level (*n =* 4). **C** Schematic of experimental procedures of ATRA oral gavage and behavioral tests. The three treatment groups include: WT + olive oil (WT + Veh); *Wdr62*^+/−^ + olive oil (*Wdr62*^+/−^ + Veh); *Wdr62*^+/−^ + ATRA (*Wdr62*^+/−^ + RA). **D** Time spent investigating Stranger I (S1) *versus* empty cage (**E**) was compared in the three groups.** E** Graph showing a significant treatment effect in the social interaction test. **F** Time spent investigating S1 *versus* Stranger 2 (S2) compared among the three groups. **G** Social preference index indicates a significant treatment effect on the social novelty test. **H** RA oral gavage significantly reduces self-grooming time in *Wdr62*^*+/−*^ mice. **I** The OFT showed no significant anxiety behavior following RA treatment in *Wdr62*^+/−^ mice. All group *n =* 10 (**D–G**). WT + Veh, *n =* 10; *Wdr62*^+/-^ + Veh, *n =* 9; *Wdr62*^+/-^ + RA, *n =* 10 (**H, I**). All data are presented as the mean ± SEM; ****P < 0.0001; ***P < 0.001; **P < 0.01; *P < 0.05; ns *P >*0.05. One-way ANOVA with the Bonferroni *post hoc* test (**B**), two-way ANOVAs with Bonferroni’s multiple comparisons tests (**D, F)**, two-tailed unpaired *t*-test (**E, G–I**).

ASD is clinically and genetically heterogeneous, and genetic screening has identified two heterozygous missense mutations of *WDR62* in patients with ASD [12]. Interestingly, the same *de novo* mutation of WDR62, p.R1452W, was also discovered to be associated with developmental disorders [41]. The predicted protein structure showed that mutation of WDR62 R650 is located at the N terminal, whereas R1452 is located at the C terminal (Fig. 6A). To investigate the effects of the mutations on WDR62 function, we overexpressed these two mutants in HEK293T cells. The protein levels of these two WDR62 mutants were significantly lower than WT WDR62 (Fig. 6B). This suggested that the ASD-associated mutations of WDR62 might affect the stability of the WDR62 protein. Considering that the WDR62 mutations associated with autism are heterozygous, we examined ASD-related behaviors in heterozygous WDR62 mutant mice. Consistently, both *Wdr62*^+/−^ and *Wdr62*^flox/+^; Nex-Cre mice displayed significant impairment in social interaction (Fig. S6A, B, E, F) and social novelty in the three-chamber test (Fig. S6C, D, G, H). Our results suggested deficits in social ability and social novelty upon loss of one copy of *Wdr62* in mice.

Growing evidence indicates that RA signaling and its responsive genetic elements regulate dendritic spine formation as well as social interaction behavior [13, 16, 42, 43]. We have shown previously that WDR62 is required for RA-mediated meiosis *via* the activation of c-jun N-terminal kinase signaling [21]. To explore the role of RA signaling in *Wdr62* deficiency-associated ASD-like behavior, *Wdr62*^*+/−*^ mice were randomly assigned into two groups for oral gavage of vehicle (olive oil) or all-trans RA (ATRA, 3 mg/kg for 5 consecutive days per week) for 2 weeks before behavioral testing according to a previous study [16] (Fig. 6C). In the social interaction test, ATRA administration was sufficient to alleviate the sociability (Fig. 6D, E) and social novelty defects of *Wdr62*^*+/−*^ mice (Fig. 6F, G). In addition, the RA-treated group spent significantly less time self-grooming than the controls (Fig. 6H). Chronic RA administration has been reported to induce anxiety-like behaviors in mice [44]. *Wdr62*^*+/−*^ mice treated with RA for 2 weeks spent a similar amount of time in the center area during the OFT, suggesting that there were no marked changes in anxiety level (Fig. 6I).

### Repletion of RA Reverses Cortical Transcriptional Changes and Alleviates Synapse Defects Associated with WDR62 Deficiency

**Fig. 7:**
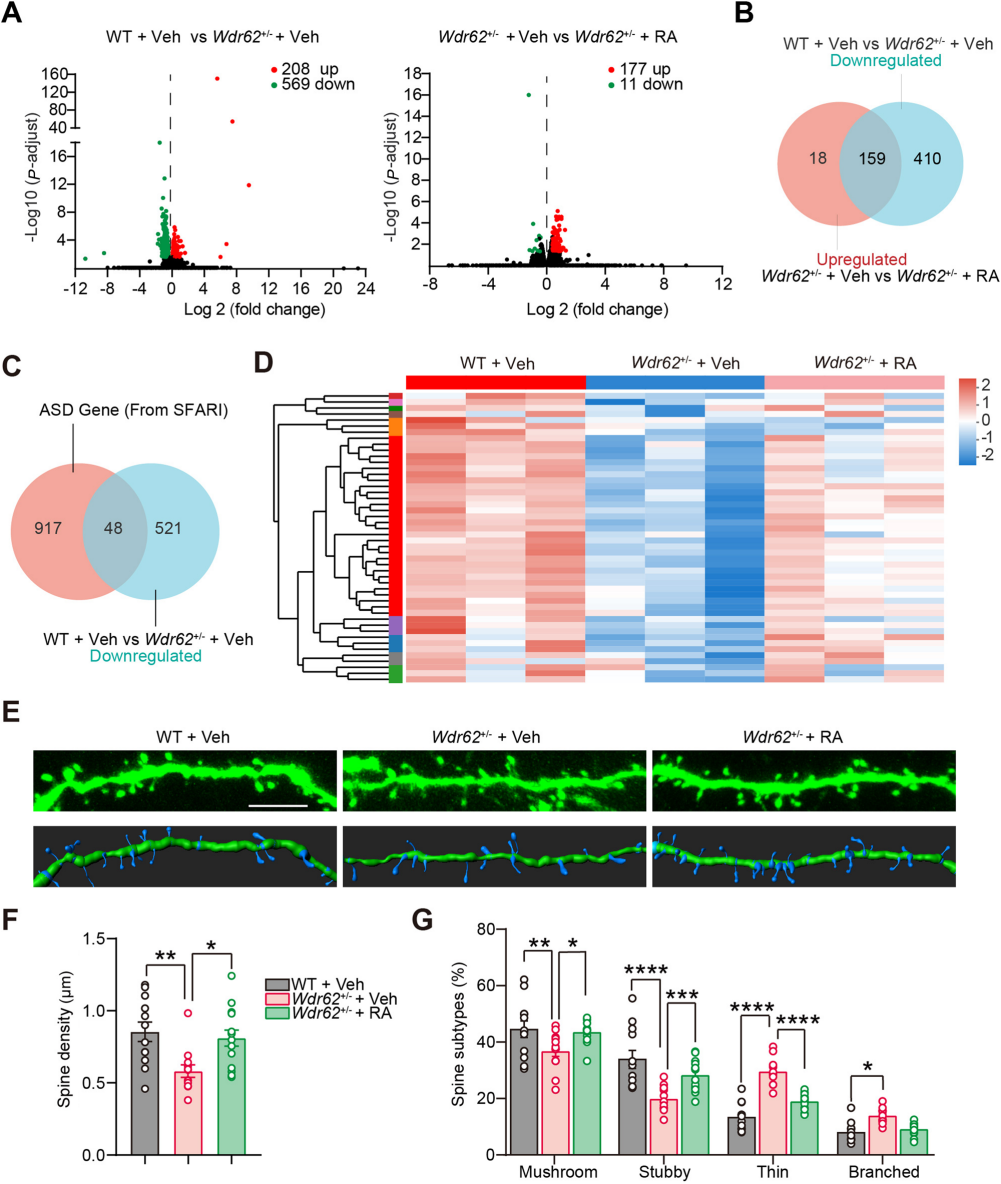
RA supplementation partially reverses cortical transcriptional changes in *Wdr62*^+/-^ mouse cortex. **A** Volcano plot representing differentially-expressed genes, which are defined as genes with p-adjust <0.05, Log 2 (fold change) >0.4. Red: upregulated genes; blue: downregulated genes. **B** Venn diagram showing the commonly regulated genes as indicated. **C** Venn diagram showing the ASD-related genes as indicated. **D** Heatmap depicting the expression patterns of ASD- related genes in the three groups. Red represents the upregulated genes and green represents the downregulated genes. Expression levels of −2.0 to 2.0 are indicated. **E** Representative images of basal dendritic spines from Thy1-*GFPm*-labeled secondary dendrites of layer 2/3 pyramidal neurons as indicated. **F** Quantification of a total number of basal spine per μm in **E**. **G** The proportion of basal dendritic spines that were mushroom (mature), stubby (mature), thin (immature), and branched (immature) subtypes. WT + Veh, *n =* 12; *Wdr62*^+/−^ + Veh, *n =* 12; *Wdr62*^+/−^ + RA, *n =* 14 neurons from 3 independent mice (**F–G**). All data are presented as the mean ± SEM; ****P <0.0001; ***P <0.001; **P <0.01; *P <0.05. One-way ANOVA with Bonferroni *post hoc* test (**F**), Two-way ANOVA with Bonferroni’s multiple comparisons test (**G**). Scale bar, 5 μm (**E**).

To investigate the potential molecular mechanism of ASD-related behavior caused by WDR62 deficiency, we compared the transcriptomes of the cortex between WT and *Wdr62*^+/−^ mice receiving oral administration of either vehicle (olive oil) or all-trans RA. A total of 777 differentially-expressed genes (DEGs) [*P*_adjust_ <0.05_,_ Log 2 (fold change) >0.4; including 208 upregulated and 569 downregulated genes] were identified between WT and *Wdr62*^+/-^ mice receiving oral administration of vehicle (Fig. 7A and Table S1). Gene Ontology (GO) analysis by meta-scope tools showed that the repressed genes were enriched in the functional terms of the gamma-aminobutyric acid signaling pathway, cell division, and chromatin organization, which are associated with ASD (Fig.S7A). In addition, the downregulated genes in *Wdr62*^+/-^ mice were enriched in cytoskeleton-dependent intracellular transport and axon, which may mediate the dendritic synaptic phenotypes (Fig. S7A). Notably, in comparison of the transcriptomes between *Wdr62*^+/-^ mice receiving oral administration of either vehicle or ATRA, 177 genes were significantly upregulated and only 11 genes were downregulated (Fig. 7A and Table S2). GO analysis indicated many genes in several functional terms involving downregulated genes that were upregulated when treated with ATRA (Fig. S7B). Synapse, pericentriolar material. and microtubule-associated functional terms were significantly enriched after RA supplementation in *Wdr62*^+/−^ mouse cortex (Fig.S7B). Surprisingly, comparing the downregulated genes induced by WDR62 haploinsufficiency and the upregulated genes after RA, 159 genes were shared (Fig. 7B). GO analysis showed that these 159 genes were involved in the regulation of the microtubule-based process, synapse assembly, chromatin organization, and pericentriolar material (Fig. S7C). We went on to inspect genes associated with ASD. Among the 569 downregulated genes in *Wdr62*^+/−^ mouse cortex, 48 genes were associated with autism according to the SFARI (Simons Foundation Autism Research Initiative) autism database (Fig. 7C). Intriguingly, the expression profile of 48 autism-associated DEGs in *Wdr62*^+/−^ mice was partially reversed in *Wdr62*^+/−^ mice brains with RA treatment (Fig. 7D). Notably, some RA signaling pathway genes, such as *Rora*, were down-regulated in *Wdr62*^+/−^ mouse cortex and were significantly up-regulated after RA supplementation (Table S3). Consistent with the transcriptomic analysis, RA treatment attenuated synapse density and maturation defects in *Wdr62*^+/−^ mouse cortical neurons (Fig. 7E–G). Collectively, our results indicated that 2 weeks of supplementation with RA was sufficient to alleviate the social behavior phenotypes in adult *Wdr62*^+/−^ mice, very likely due to the complementation of the of expression many genes, especially the ASD-associated genes.

## Discussion

### Association between WDR62 and ASD

Mutations or deficiency of *WDR62* causes many diseases in humans, such as neurological disorders, infertility, and tumorigenesis [45]. Recently, *WDR62* heterozygotic *de novo* mutations were identified in ASD patients by genetic screening; however, animal model validation was lacking [12]. This study aimed to explore the relationship between WDR62 disruption and mouse behaviors, as well as neuronal development to ascertain whether WDR62 alterations have relevance to ASD.

We examined developmental and behavioral phenotypes in different lines of *Wdr62*-KO mice, which have global developmental delays with impaired cognitive and social interaction functions. While mice with WDR62 deficiency in post-mitotic neurons have no global developmental phenotypes but are defective in social behavior. This is consistent with recent findings that some genes impart risk to both ASD and Neurodevelopmental disorders (NDD) [46]. The defects in social interaction and impaired learning and memory in *Wdr62* KO may be associated with reduced brain size. However, neuron-specific conditional *Wdr62*-cKO mice which have normal brain sizes, also exhibit impaired social interactions and increased repetitive behaviors. This demonstrates that the ASD-related behaviors caused by WDR62 disruption are not necessarily associated with brain size. This situation has also been found in Zika virus (ZIKV)-infected patients and mouse models. Abnormal social behaviors have been reported in mice infected with ZIKV several days after birth that have normal brain sizes [47]. Another study in MECP2 duplication mice showed a significant social recognition deficit while learning and memory were comparable in MECP2 duplication and wild-type mice [48]. Of course, we cannot rule out the influence of the sensitivity of different behavioral tests.

### WDR62 Regulates Synapse Development

Several groups have established that *Wdr62* mutant/KO mice have a reduction in brain size that is due to the disrupted development of neural progenitor cells [22, 27, 28, 49]. However, the function of this genetic alteration in postnatal and adult animals was not clear. Our neuron-specific *Wdr62*-cKO mice had a generally normal brain size and structure, yet exhibited ASD-like behaviors. These prompted us to explore the function of WDR62 apart from embryonic neurogenesis. The structure and function of neurons and synapses are important for transmitting signals between neurons. Reduced neuronal complexity among the pyramidal neurons of both layers 2/3 and 5 in the cortex was detected in *Wdr6*2 Nex-cKO mice. Detailed analysis indicated that the dendritic spine density of pyramidal neurons was also significantly reduced. In addition, the reduced frequency of mEPSCs in *Wdr62*-cKO neurons was consistent with the alterations in the synapse. These findings suggest that the developmental defects of neuronal dendrites and spines, as well as dysfunction of the synapse, may contribute to ASD-like behaviors in WDR62-deficient mice. The spine density and morphology of layer 5 pyramidal neurons in the motor cortex were significantly changed in *Wdr62* Nex-cKO mice. This is consistent with the clinical studies in patients with WDR62 mutations who have motor delays. However, the rotarod test indicated *Wdr62-*Nex-cKO mice had no defects in locomotor activity. ASD patients always make the same movement. Interestingly, we noted that a small population of heterozygous *Wdr62*-KO mice exhibited a higher frequency of repetitive circular locomotion, which indicated autism-like behavior. Whether WDR62 deletion affects other fine movements requires further testing.

Excitation-inhibition (E/I) imbalance is considered to be an important pathophysiological mechanism underlying ASD [50]. WDR62 haploinsufficiency resulted in the downregulation of genes involved in the GABA signaling pathway (Fig. S7A). We analyzed the parvalbumin-positive (PV^+^) cells in the adult sensory cortex of WT and *Wdr62*^+/−^ mouse brains. There was a subtle decrease in the number of PV^+^ cells in *Wdr62*^+/−^ mouse cortex, but with no significant differences (Fig. S8A). Consistent with this, the PV^+^ cells were not significantly changed in *Wdr62*-Nex-cKO mouse cortex (Fig. S8B). However, whether WDR62 deficiency impaired E/I balance and conditional deletion of *WDR62* in the inhibitory circuit will contribute to autism-associated behavior still needs to be explored.

### RA Supplementation as a Potential Therapeutic Strategy for ASD

The brain is more efficient at converting vitamin A to RA to activate RA receptors than other tissues [14]. More importantly, RA supplementation has been shown to be effective in alleviating social impairments not only in animals but also in ASD patients [18, 19]. RA oral gavage in mice with WDR62 haploinsufficiency significantly reverses the defects in social interaction and repetitive behavior, providing further support for RA as a potential therapy. Importantly, we found that RA partially reversed the transcriptional changes in the *Wdr62*^+/−^ mouse brain, especially the 48 ASD-associated genes.

Understanding how genetic modification affects brain development and behavior would improve the diagnosis, treatment, and prevention of disease. Our study with different *Wdr62*-KO mouse lines indicates that abnormal social behaviors caused by WDR62 deficiency are not necessarily associated with reduced brain size or learning and memory deficits.

## Supplementary Information

Below is the link to the electronic supplementary material.

Supplementary file1 (PDF 1240 kb)
