# Supplementary material for: WDR62-deficiency Causes Autism-like Behaviors Independent of Microcephaly in Mice
Source: Neurosci Bull. 2022 Dec 26;39(9):1333–47. doi: 10.1007/s12264-022-00997-5 (PMC10465473; doi:10.1007/s12264-022-00997-5)
Supplement: Supplementary file 1 — Supplementary file1 (PDF 1240 kb) [file 12264_2022_997_MOESM1_ESM.pdf]

Supplementary materials

Supplementary Figures

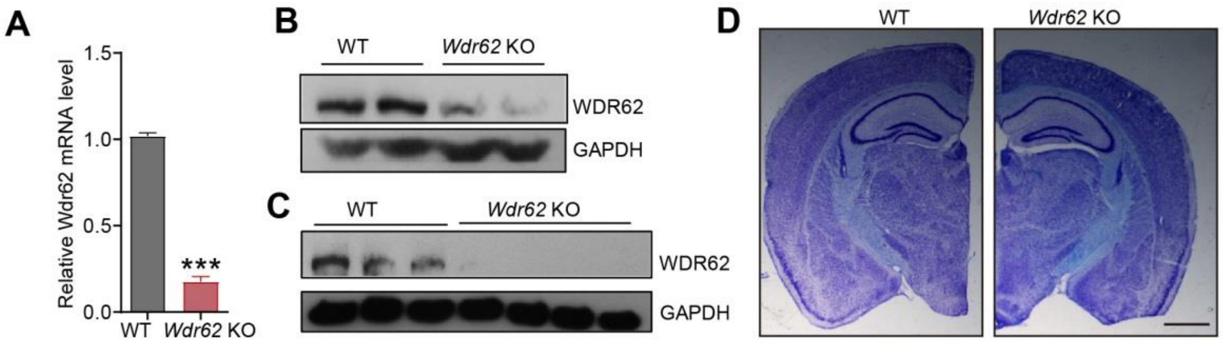

Fig. S1 The knockout efficiency of *Wdr62*-KO mice (related to Figure 1). **A** Real-time PCR analysis of *Wdr62* mRNA levels in E18.5 mouse brains (WT n = 2, *Wdr62*-KO n = 4). Data are presented as the mean ± SEM, ****P* <0.001, two-tailed unpaired *t*-test. **B** Immunoblots showing WDR62 protein levels in P6 WT and *Wdr62*-KO mouse brains. **C** Western blots showing WDR62 protein expression in primary cultured MEFs after 2 days in vitro. Glyceraldehyde-3-phosphate dehydrogenase (GAPDH) protein level was loaded as a control. **D** Nissl staining show a thinner cortex in 2-month-old *Wdr62*-KO mouse brain, while the general morphology (different layers) does not change. Scale bar, 1 mm.

Fig. S2 *Wdr62* expression in Nex-cKO mice (related to Figure 3). **A** Relative *Wdr62* mRNA levels

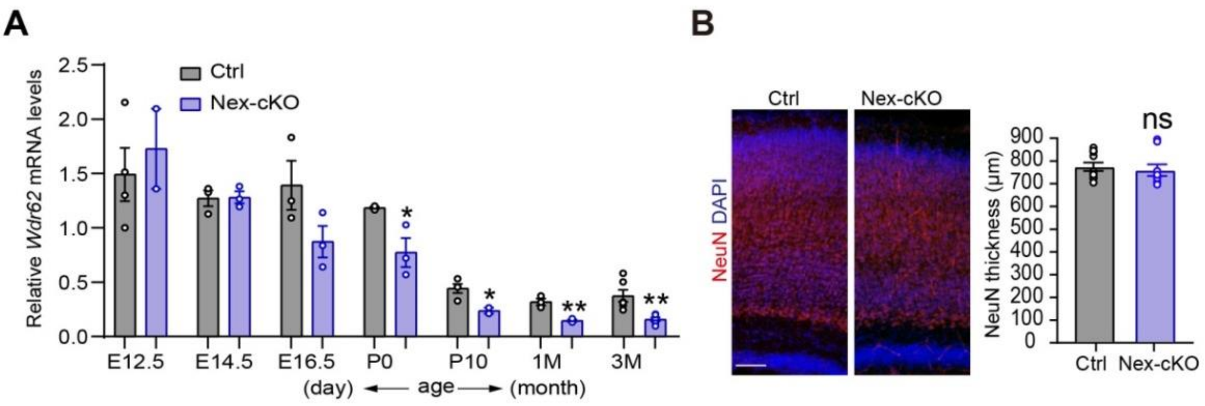

during different developmental stages of *Wdr62*$^{\text{folx/flox}}$; Nex+/+ (control, ctrl) and *Wdr62*$^{\text{folx/flox}}$; Nex Cre/+ (*Wdr62*-Nex-cKO) littermate mouse brains. E12.5: Ctrl n = 4, *Wdr62*-Nex-cKO n = 2; E14.5, E16.5, P0 and 1-month-old mice: Ctrl n = 3, *Wdr62*-Nex-cKO n = 3; P10 mice: Control n = 4, *Wdr62*-Nex-cKO n = 2; 3-month-old mice: Ctrl and *Wdr62*-Nex-cKO n = 6. **B** Coronal sections of Ctrl and *Wdr62*-Nex-cKO mice at P3 immunostained for the newborn neuronal marker NeuN and nuclei using 4',6-diamidino-2-phenylindole (DAPI). Bar graph shows the density of NeuN+ cells in the cortical plate (Ctrl n = 10, *Wdr62*-Nex-cKO n = 9). n = slices from at least three independent brains. Data are presented as the mean ± SEM; **P <0.01, *P <0.05, ns P >0.05; multiple unpaired t-tests (**A**); Mann-Whitney test (**B**). Scale bar, 100 μm.

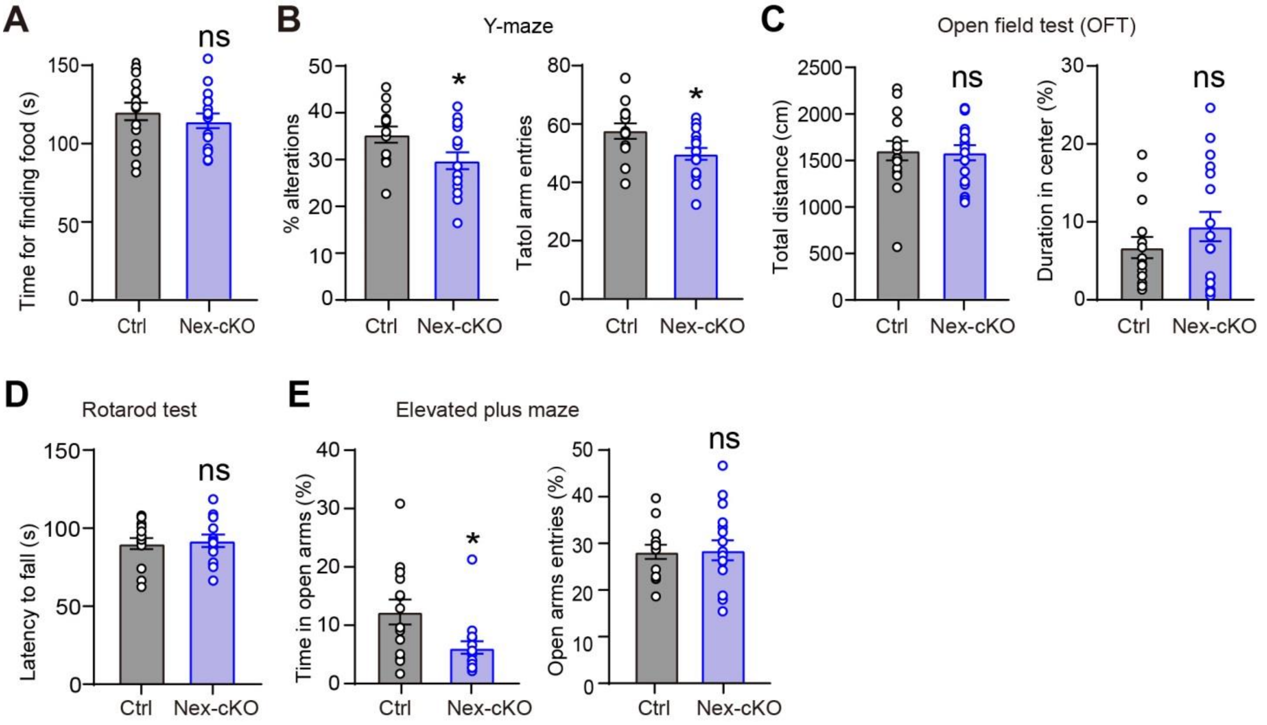

Fig.S3 *Wdr62*-Nex-cKO mice have a normal acoustic startle response, locomotor activity, and anxiety behavior (related to Figure 3). **A** *Wdr62*-Nex-cKO mice show no significant difference from control littermates in time taken to find food. **B** Left: Y maze spontaneous alternation test shows fewer spontaneous alternations in *Wdr62*-Nex-KO mice than those in control mice. Right: Y maze total arm

entries show cKO *Wdr62*-Nex-KO mice have fewer total arm entries than control mice. **C** The OFT indicates normal locomotor activity. **D** The rotarod test shows normal ability and motor learning in *Wdr62*-Nex-cKO mice (Ctrl $n = 15$, *Wdr62*-Nex-cKO $n = 17$). **E** In the elevated plus-maze test, *Wdr62*-Nex-cKO mice spend less time in the open arm, which indicates an anxiety tendency (Ctrl $n = 15$, *Wdr62*-Nex-cKO $n = 17$). All data are presented as the mean ± SEM. ***P <0.001; **P <0.01; *P <0.05; ns P >0.05; two-tailed unpaired *t*-tests (**A–C** and **E**); Mann-Whitney test (**D**).

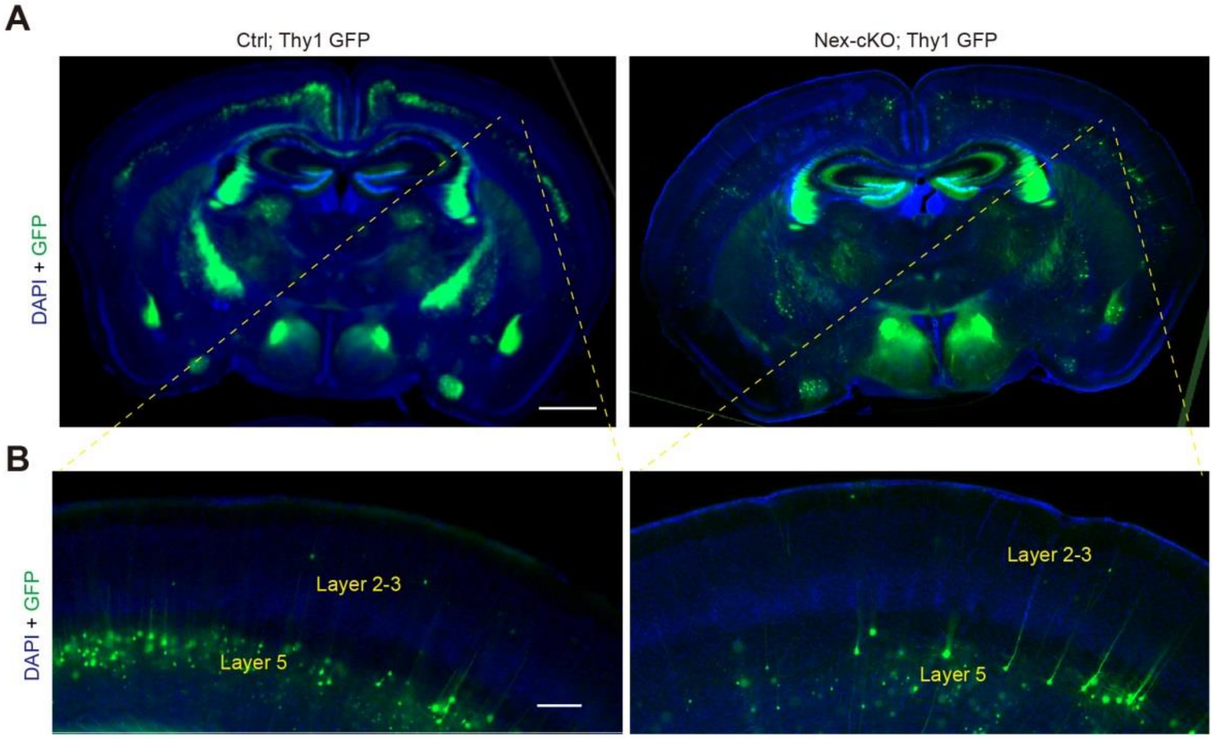

Fig. S4 Thy1-GFPm-labeled pyramidal neurons in the brain (related to Figure 4). **A** Large scale images of Thy1-GFPm-labeled control and *Wdr62*-Nex-cKO mouse brain slices. Scale bar, 1 mm. **B** Image showing the region (layers 2/3 and 5) of analysis in the brain. Scale bar, 250 μm.

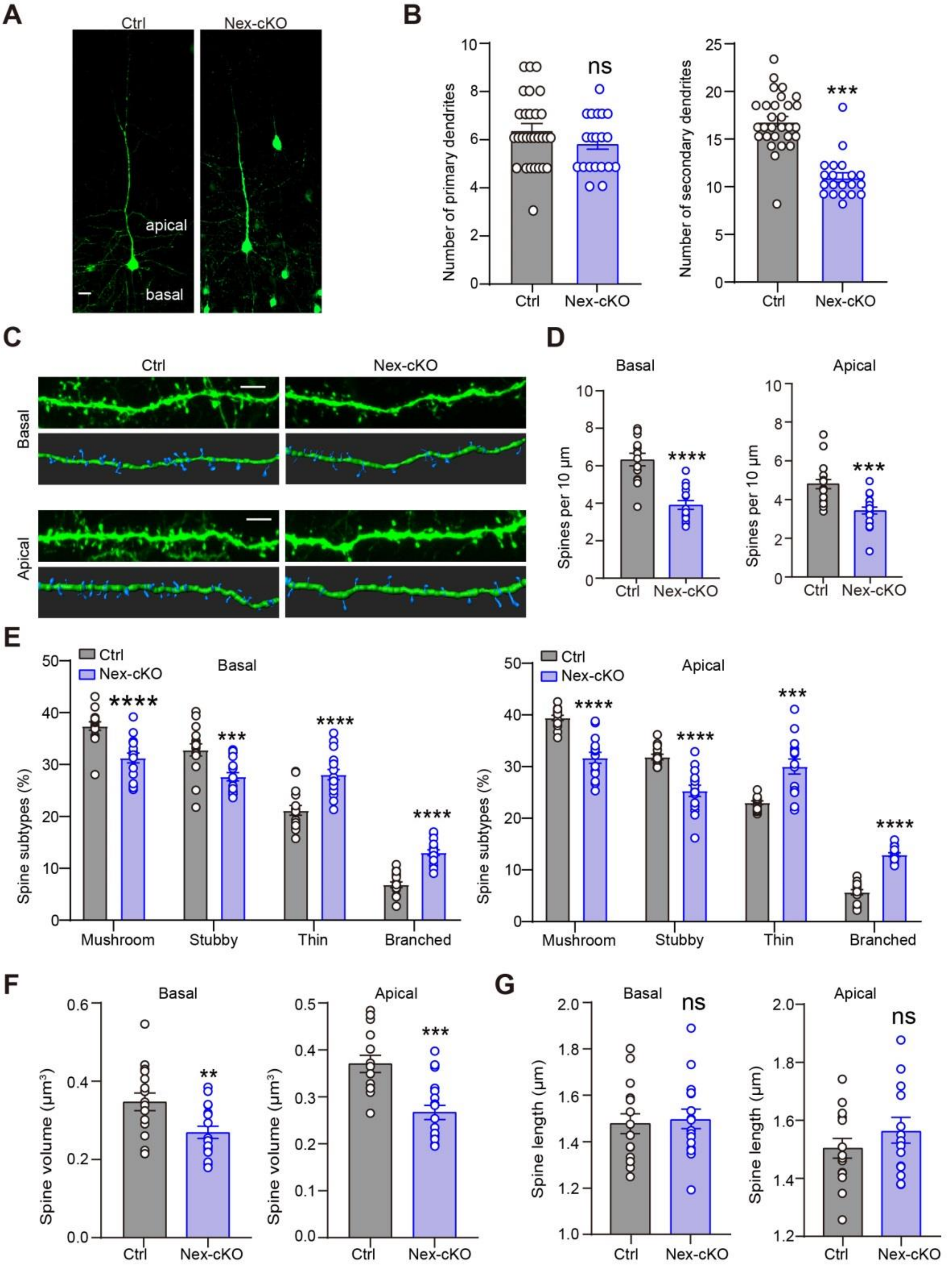

Fig.S5 Morphology of layer 5 pyramidal neurons in the cortex (related to Figure 5). **A** Representative

images of Thy1-GFPm-labeled layer 5 pyramidal neurons in the cortex. **B** Quantification indicates reduced secondary dendritic arborization in *Wdr62*-deficient neurons. **C** Higher-magnification images of apical/basal dendritic spines from Thy1-GFPm-labeled secondary dendrites of layer 5 pyramidal neurons. **D** Quantification of total apical/basal dendritic spine density in **C**. **E** The proportion of apical/basal dendritic spines that are mushroom (mature), stubby (mature), thin (immature), and branched (immature) subtypes; Ctrl n = 16 cells from three mice, *Wdr62*-Nex-cKO n = 17 from three mice. **F, G** Quantification shows that the volume, but not the length, of apical/basal dendritic spines is significantly reduced. All data are presented as the mean ± SEM; ****P <0.0001, ***P <0.001; **P <0.01; *P <0.05; ns P >0.05; two-way ANOVA with Bonferroni's multiple comparisons test (**E**), two-tailed unpaired t-tests (**D, F**, and **G**). Scale bar, 5 µm.

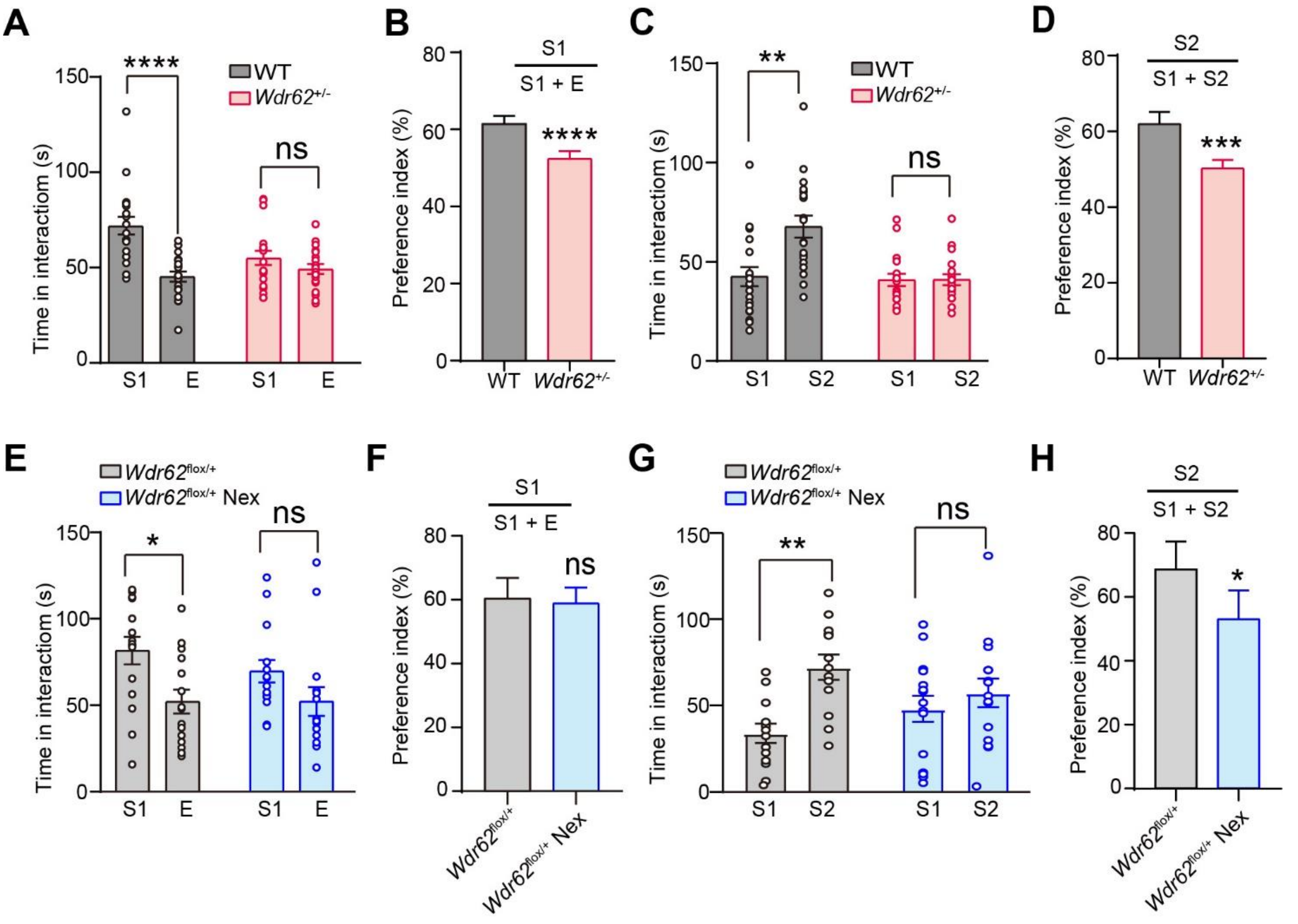

Fig.S6 WDR62 haploinsufficiency results in social interaction defects (related to Figure 6). **A** *Wdr62*$^{+/–}$ mice show a shorter social interaction time with (stranger 1) S1 than control (Ctrl) mice. **B** Graph of social preference index (% time spent investigating social or empty cage out of total object investigation time) showing a significantly reduced preference index of *Wdr62*$^{+/–}$ mice during the social interaction test. **C** In the social novelty test, *Wdr62*$^{+/–}$ mice display no preference for interacting with the (stranger 2) S2 or S1 mouse. **D** The preference index for social novelty is significantly reduced in *Wdr62*$^{+/–}$ mice. **E** *Wdr62*$^{flox/+}$; Nex-Cre mice show a shorter social interaction time with S1 than control mice. **F** Graph of social preference index showing normal preference index during the social interaction test. **G** In the social novelty test, *Wdr62* $^{flox/+}$; Nex-Cre mice display no significant preference for interacting with the S2 or S1 mouse. **H** The preference index of social novelty is reduced in *Wdr62* $^{flox/+}$; Nex-Cre mice. All data are presented as the mean ± SEM; ****P <0.0001; ***P <0.001; **P <0.01; *P <0.05; ns: P >0.05; WT and *Wdr62*$^{+/–}$ n = 19 (**A–D**). Ctrl and *Wdr62*$^{flox/+}$; Nex-Cre n = 15 (**E–H**). two-way ANOVAs with Bonferroni's multiple comparisons test (**A, C, E**, and **G**), two-tail unpaired t-tests (**B, D, F,** and **H**).

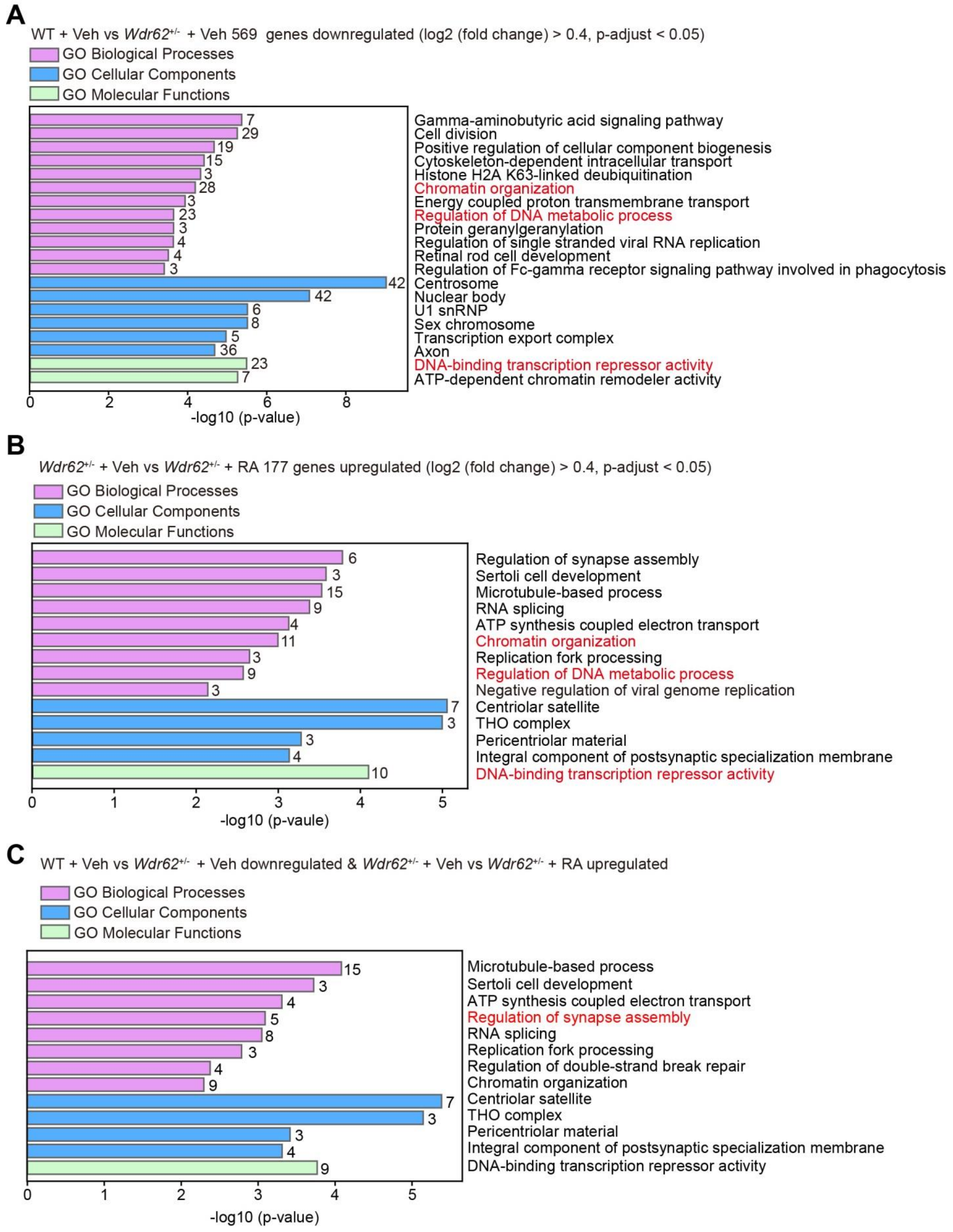

Fig.S7 (related to Figure 7). **A** Gene ontology (GO) analysis reveals the GO terms indicating the biological processes, cellular components, and molecular functions enriched in downregulated genes

between WT mice receiving oral administration of vehicle (WT + Veh) and *Wdr62*$^{+/-}$ mice receiving oral administration of vehicle (*Wdr62*$^{+/-}$ + Veh). **B** GO analysis reveals the GO terms indicating the biological processes, cellular components, and molecular functions enriched in upregulated genes between *Wdr62*$^{+/-}$ mice receiving oral administration of vehicle (*Wdr62*$^{+/-}$ + Veh) and *Wdr62*$^{+/-}$ mice receiving oral administration of ATRA (*Wdr62*$^{+/-}$ + RA). **C** GO analysis of the common regulatory gene in Figure 7B. Terms indicating the biological processes, cellular components, and molecular functions enriched in downregulated genes between WT mice receiving oral administration of vehicle (WT + Veh) and *Wdr62*$^{+/-}$ mice receiving oral administration of vehicle (*Wdr62*$^{+/-}$ + Veh).

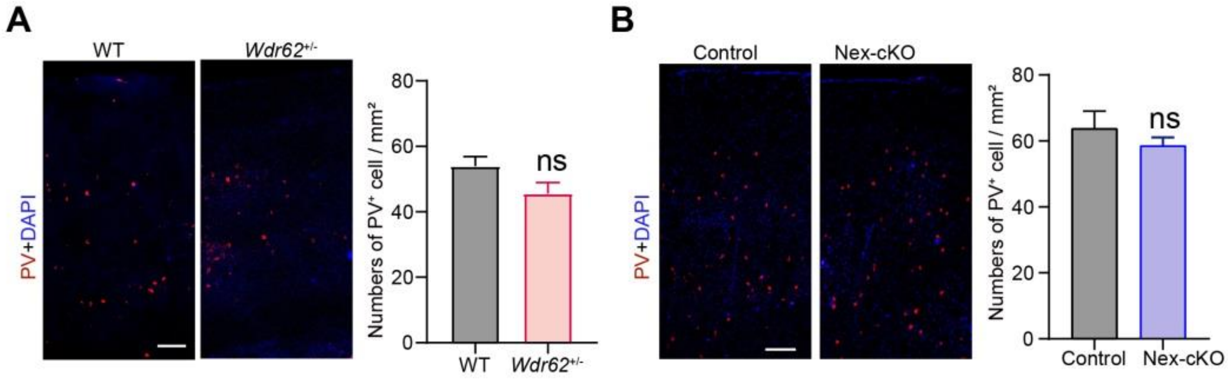

Fig. S8 (related to Figures 3 and 7). **A, B** Left: Coronal sections of two-month-adult *Wdr62*$^{+/-}$ and WT littermates or *Wdr62* Nex-cKO and littermate control mice; the cerebral cortex is stained with antibodies of parvalbumin (PV, inhibitory neuron marker). Nuclei are labeled with DAPI. Right: Quantification of numbers of PV-positive inhibitory neurons on the left. WT and *Wdr62*$^{+/-}$ $n = 8$ (A); Control $n = 4$, *Wdr62* Nex-cKO $n = 5$ (B). n = brain slices from at least three mice. All data are presented as the mean ± SEM; unpaired *t*-tests; ns $P > 0.05$. Scale bar, 100 μm.

Supplemental methods

Behavioral Study

Animals

Mice were housed under standard laboratory conditions on a 12-h dark/light cycle, and all behavioral testing was carried out during the light phase. The generation of mice carried a *Wdr62*^{floxed} allele with a deletion of *Wdr62* exon 2 and a reading frame shift in exon 3 [1]. To generate mice carrying a universal null allele of *Wdr62* (*Wdr62*-KO), *Wdr62* floxed mice were crossed with *ZP3-Cre* mice. To conditionally inactivate the *Wdr62* gene in mice, *Wdr62*$^{flox/flox}$ mice were crossed with Nex cre/+ mice to obtain *Wdr62*$^{flox/flox}$; Nex-Cre (*Wdr62*-Nex-cKO) mice. Mice were genotyped using the polymerase chain reaction (PCR) of DNA extracted from a neonatal toe biopsy. Primers are listed in Table S4. For most experiments, the resulting comparison littermates were analyzed. Male *Wdr62* KO and littermate control mice were used for all of the experiment assays of this genotype. Male and female (male:female ratio 1:1) *Wdr62*$^{+/-}$ and littermate control mice generated by *in vitro* fertilization were examined at 2 months for RA treatment, behavioral, morphological and RNA-seq analysis and no significant sex-dependent differences were found. All available male and female *Wdr62*-Nex-cKO and littermate controls were used accordingly for experiments including behavioral, electrophysiological, and morphological studies, and no significant sex-dependent differences were found.

Open-field Test

The test mouse was first allowed to explore the open-field test (OFT) apparatus for 5 min. After washing the apparatus with 75% ethanol, the test mouse was introduced into the apparatus again and allowed to explore. The time spent in the center region (25%) of the apparatus and the distance traveled in the apparatus were calculated.

Y Maze Test

The Y maze test is a rapid and easily-administered test to assess spatial working memory. A Y maze with a three-armed runway (36 × 6 × 12 cm; at a 120_ angle from each other) was placed in the test room. Different visual cues were located on the wall at the end of each arm. Activity was recorded using a camera, and behavior was assessed using automatic video tracking software, Smart 3.0 (Panlab Co.). Each mouse was randomly introduced into the end of one arm, facing the center, and allowed to freely explore the apparatus for 5 min. The equipment was cleaned with 70% ethanol and dried before a new trial started. Mice entered a different arm when they remembered which arm it had entered on a previous trial. A point was given when the mouse entered each of the three arms without repetition (e.g., CAB, BCA, and ABC). The number of points was then divided by the total arm entries minus one.

Radial Arm Maze Task

The radial arm maze is a paradigm that is used to assess working and reference memory in rats and mice. Before the task, mice were allowed 1 week to habituate to the testing room and fasted for 24 h. After every training day, restricted food was provided. For the working memory assessment, all arms contained a reward (food), and the animal was required to visit each arm once only. To assess reference memory, only some of the arms contained a reward, and the animal was required to visit only the baited arms. Visits to the same or non-baited arms counted as working memory or reference memory errors, respectively.

Novel Object Recognition

Novel object recognition is a relatively sensitive test for assessing cognitively-enhanced activity [1]. An open-field box (36 × 36 × 25 cm^3) was used for this test. During the training period, two identical novel objects were placed in the box, and mice freely explored the box for 5 min. After 1 day, one of

the familiar objects used during the training session was replaced with a new object, and the mouse was placed back into the open-field box for 5 min. The time each mouse spent exploring objects during the training and test phases was recorded using automatic video tracking software. The ratio of time spent exploring the novel object to that spent exploring both objects was used as a measure of recognition memory.

Three-chamber Test

The three-chamber test is a simple social interaction test, in which behaviors are video-recorded and analyzed to assess the active interaction time between test and novel mice [2]. It is a sensitive method for examining autism-related behavioral deficits and has been used to measure autism-like behaviors in mutant mouse models [3-5]. Briefly, the arena consisted of a transparent box, which was divided into three chambers of the same size with removable doors in each chamber. The test mouse and the age/sex-matched C57BL/6 WT mouse (not littermates) were kept in the test room individually 1 h before the experiment. Two wire cups were placed in the corner of the left and right chambers. The test mouse was introduced into the central chamber and was allowed access to the three chambers for 10 min. During the first stage, a novel age/gender-matched C57BL/6 unfamiliar mouse (Stranger 1—'S1') was introduced into the wire cup of the left or right chamber, and an empty wire cup (Empty, 'E'). The test mouse was allowed access to the three chambers and both wire cups for 10 min. The time the test mouse spent interacting with S1 or E was recorded. The chamber was cleaned with 75% ethanol after each test. During the second stage, the empty wire cage was replaced with another novel age/gender-matched C57BL/6 WT mouse (Stranger 2—'S2'). The test mouse was allowed access to the three chambers and both wire cups for 10 min. The time the test mouse spent interacting with the S1 or S2 was recorded. No position bias was found. The preference index for each animal was

calculated as: S1/(S1 + E) × 100% or S2/(S1 + S2) × 100% accordingly [6].

Self-grooming Test

The mice were first habituated in a clean chamber individually for 10 min. The time spent self-grooming was recorded for 10 min. Self-grooming included actions of face-wiping, scratching of the head, neck, or ears, and full-body grooming.

Stranger-intruder Test

The stranger-intruder test is a simple method to directly assess sociability. Each test mouse was placed into a new cage with clean bedding. A C57 male mouse, matched for weight and age, was placed in the same cage and left for 10 min. The time the test mouse actively sniffed the C57 mice was recorded.

Fear-conditioning Task

In this test, a freezing response, which is considered a reliable measure of fear in mice, was elicited by unconditioned electric shock stimuli that were matched with conditioned stimuli of sounds or the surrounding context [7]. The apparatus consisted of a conditional shock chamber (25 × 25 × 25 cm^3), surrounded by a large metal lockable box to minimize noise from the environment and control the recording software, PACKWIN 2.0 (Panlab). The conditional stimulus (CS) was an 80-dB sound at 2000 Hz for 28 s, and the unconditional stimulus (US) was a foot shock at 0.5 mA that lasted 2 s. During the test, the background white noise was 60 dB. On day 1, mice were allowed to freely explore the shock chamber for 2 min. Then, the CS was presented, which was followed by the US. The CS and US were repeated three times. There was a 30-s interval after each CS-US pairing. Mice were introduced into the same contextual chamber 24 h later. The same procedure was applied, except the CS paired with the US was turned off. After 48 h, mice were placed into the same chamber with a novel context (i.e., the chamber was changed to a different color and shape). The same procedure was

applied, except the US was turned off. Animals were observed for freezing behavior every 30 s in each condition. The percentage of freezing bouts was recorded.

Elevated Plus-maze

The elevated plus-maze was used to investigate the anxiety level of mice and comprised two open arms (30 × 6 cm^2), two closed arms (30 × 6 cm^2), and a center section (6 × 6 cm^2). The same arms were on the opposite sides of the center section. The mouse was placed in the center section of the maze with its head facing the same closed arm at the start of the experiment. The behavior of mice in the elevated plus-maze was recorded and analyzed for 10 min. The time the mouse spent in open or closed arms was exported by the software.

Morris Water Maze Test

The water maze test consisted of a circular pool (120 cm in diameter and 50 cm in height), a platform (6 cm in diameter), a camera fixed above the pool, and a computer connected to the camera. The pool was filled with water at ~22°C and 25 cm deep. The platform was placed 1 cm underwater, and a layer of non-toxic titanium dioxide floated on the water surface to prevent animals from seeing the underwater platform. Brightly colored cardboard or plastic panels of different shapes were attached to the walls of the pool as markers for the spatial orientation of the animals. The experiment consisted of two phases: the place navigation and spatial probe experiments. During the training period (5 days), the platform was placed in one quadrant of the pool, and mice were placed into the water facing the wall from four entry points at a fixed time each day. The mice were then quickly dried with towels and placed under a 37°C heat lamp to maintain body temperature. On day 6, the spatial exploration test was conducted by removing the platform after the place navigation test, placing the mouse in the pool in the quadrant opposite to the previous platform, and recording their swimming trajectory for 60 s to

examine the mouse's memory of the original platform. A video tracking system was used to record the swimming path of each mouse in the pool, and the ratio of the time spent within the quadrant where the original platform was located for 60 s to the time spent in the other three quadrants was calculated.

Nest Building Test

The mice were separated and kept in single cages 1 h before lights out. A 3.0 g square (10 × 10 cm^2) of skimmed cotton was placed in the cage. After 12 h, mice were observed for nesting, and their genotypes were photographed and scored according to scoring criteria: 0–1: 90% of the skimmed cotton remained intact; 1–2: a small portion of the skimmed cotton was torn but most remained intact (50–90%); 2–3: most of the skimmed cotton was torn into pieces (50–90%) but no nesting; 3–4: >90% of the skimmed cotton was torn into pieces and formed a flat nest (<50% of the height of the mice); 4–5: >90% of the skimmed cotton was torn into pieces, forming a high-quality nest (>50% of the height of the mice). Because of the complexity of the experiments, scores could be decimals. For example, if the nest was made perfectly, but 10% of the skimmed cotton remained unshredded, the score could be 4.5 points.

Rotarod Test

The rotarod apparatus was used to examine motor coordination. Each mouse was tested over four trials once a day for 2 days, with the rod rotation increasing from 4 to 40 rpm in 5 min. The interval between each trial was 30 min. The rotarod apparatus was stopped when the test mouse fell, and the latency to fall off the rod was determined. The average latency of the four trials on day 2 was calculated.

Underground Food Sniffing Test

The mice were fasted for 24 h the day before the test. During the task, each mouse was placed in a clean rat cage (42.5 × 26.6 × 15.5 cm^3) with 4-cm-deep bedding that contained a hidden food pellet

(0.2 g). The time required to find the buried food pellet was measured (up to 5 min).

Acute Slice Preparation

Mice-aged 2–3-week-old mice (P17 – P21 in this experiment) were anesthetized with 4% isoflurane, decapitated, and the brain was dissected. The whole brain was cut into 300-μm slices in cutting solution as described in [8], allowed to recover at 34°C for 30 min, and stored at room temperature. Solutions were continuously gassed with 95% oxygen (O_2)/5% carbon dioxide (CO_2).

Whole-cell Recordings

Whole-cell voltage-clamp recordings were acquired from the pyramidal cells of cortical layers 2/3. Pyramidal neurons were identified according to location and morphology. All recordings were made at 20-25°C. The internal solution (in mmol/L) was: 135 $CsMeSO_4$, 8 NaCl, 10 HEPES, 5 QX314-Cl, 4 Mg-ATP, 0.3 Na-GTP, 0.3 EGTA, and 0.1 spermine. Osmolarity was adjusted to 290–295 mOsm, and pH was buffered at 7.3-7.4. The external solution (mmol/L) was: 119 NaCl, 2.5 KCl, 4 $CaCl_2$, 4 $MgCl_2$, 1 NaH_2PO_4, 26.2 $NaHCO_3$, and 11 glucose, bubbled continuously with 95% O_2/5% CO_2. To record miniature excitatory postsynaptic currents (mEPSCs), picrotoxin (100 μmol/L) was added to the external solution, and the cell membrane was held at -70 mV. Current responses were collected with a Multiclamp 700B amplifier (Axon Instruments), filtered at 2 kHz, and digitized at 10 kHz. Cells with a series resistance >20 MΩ were excluded from analysis.

RNA Extraction and QPCR Analysis

Total RNA was extracted from brain tissue samples using TRIzol reagent (Invitrogen, Cat# 15596018). First-strand cDNA was generated using M-MLV reverse transcriptase (Promega, Cat# M1701) according to the manufacturer's protocols. Primers are as listed in Table S4.

RNA Sequencing and Data Analysis

Foe library preparation for transcriptome sequencing, a total amount of 3 μg RNA per sample was used as input material for the RNA sample preparations. Sequencing libraries were generated using the NEBNext® Ultra™ RNA Library Prep Kit for Illumina® (NEB, USA) following the manufacturer's recommendations, and index codes were added to attribute sequences to each sample. Briefly, mRNA was purified from total RNA using poly-T oligo-attached magnetic beads. Fragmentation was carried out using divalent cations under elevated temperature in NEBNext First Strand Synthesis Reaction Buffer (5X). First strand cDNA was synthesized using random hexamer primers and M-MuLV Reverse Transcriptase (RNase H-). Second strand cDNA synthesis was subsequently performed using DNA Polymerase I and RNase H. Remaining overhangs were converted into blunt ends *via* exonuclease/polymerase activity. After adenylation of 3' ends of DNA fragments, NEBNext Adaptor with hairpin loop structure were ligated to prepare for hybridization. In order to select cDNA fragments of preferentially 150–200 bp in length, the library fragments were purified with the AMPure XP system (Beckman Coulter, Beverly, USA). Then 3 μl USER Enzyme (NEB, USA) was used with size-selected, adaptor-ligated cDNA at 37°C for 15 min followed by 5 min at 95 °C before PCR. Then PCR was applied with Phusion High-Fidelity DNA polymerase, Universal PCR primers and Index (X) Primer. Finally, PCR products were purified (AMPure XP system) and library quality was assessed on the Agilent Bioanalyzer 2100 system. Clustering and sequencing the clustering of the index-coded samples was performed on a cBot Cluster Generation System using TruSeq PE Cluster Kit v3-cBot-HS (Illumia) according to the manufacturer's instructions. After cluster generation, the library preparations were sequenced on an Illumina Hiseq2500/X platform and 125/150 bp paired-end reads were generated. The resulting P values were adjusted using the Benjamini-Hochberg procedure. Differentially-expressed genes (DEGs) were defined according to an adjusted p-value <0.05 and Log2 (fold

change) >0.4. Gene Ontology (GO) enrichment analysis of DEGs was performed by Metascape database online analysis. GO terms with a corrected p-value <0.05 were defined as significantly enriched by DEGs.

Table S4 Primers for genotyping and QPCR analysis

**Primer**	**Sequence (5'-3')**
WDR62 Floxed mouse line – Forward	GGCAGGTTAAGCTTTGTGAGTT
WDR62 Floxed mouse line – Reverse	GGATGCTTGCCTAGTGTGTAC
WDR62-KO- Reverse	CGTCTCATCATGAAGCCTAGG
Nex-Cre mouse line – Primer1	GAGTCCTGGAATCAGTCTTTTTC
Nex-Cre mouse line – Primer2	AGAATGTGGAGTAGGGTGAC
Nex-Cre mouse line – Primer3	CCGCATAACCAGTGAAACAG
Thy1-YFP- Forward	AAGTTCATCTGCACCACCG
Thy1-YFP- Reverse	TCCTTGAAGAAGATGGTGCG
Mouse *Wdr*62 QPCR- Forward	ACCGCAACGTAAGGGTCTACACA
Mouse *Wdr*62 QPCR- Reverse	AAACATCTTGGCAACACACTCGCC
Mouse *Gadph* QPCR- Forward	TGATGACATCAAGAAGGTGGTGAAG
Mouse *Gadph* QPCR- Reverse	TCCTTGGAGGCCATGTAGGCCAT

References:

[1] Mathiasen J, DiCamillo A. Social recognition assay in the rat. Curr Protoc Neurosci 2010, Chapter 8: Unit8.5I.

[2] Crawley JN. Mouse behavioral assays relevant to the symptoms of autism. Brain Pathol 2007, 17: 448-459.

[3] Kwon CH, Luikart BW, Powell CM, Zhou J, Matheny SA, Zhang W, *et al*. Pten regulates neuronal arborization and social interaction in mice. Neuron 2006, 50: 377-388.

[4] Lijam N, Paylor R, McDonald MP, Crawley JN, Deng CX, Herrup K, *et al*. Social interaction and sensorimotor gating abnormalities in mice lacking *Dvl1*. Cell 1997, 90: 895-905.

[5] Moretti P, Bouwknecht JA, Teague R, Paylor R, Zoghbi HY. Abnormalities of social interactions and home-cage behavior in a mouse model of Rett syndrome. Hum Mol Genet 2004, 14: 205-220.

[6] Zhang K, Yu F, Zhu J, Han S, Chen JH, Wu XY, *et al*. Imbalance of excitatory/inhibitory neuron differentiation in neurodevelopmental disorders with an *NR2F1* point mutation. Cell Rep 2020, 31: 107521.

[7] Blanchard RJ, Blanchard DC. Passive and active reactions to fear-eliciting stimuli. J Comp Physiol Psychol 1969, 68: 129-135.

[8] Tao WC, Díaz-Alonso J, Sheng N, Nicoll RA. Postsynaptic δ1 glutamate receptor assembles and maintains hippocampal synapses via Cbln2 and neurexin. Proc Natl Acad Sci U S A 2018, 115: E5373-E5381.
